# Enzyme-Powered CO_2_ Utilization: A Bifunctional Immobilized Biocatalyst for Intensified CCU of Industrial Feedstocks to High-Value Chemicals

**DOI:** 10.1021/acssuschemeng.5c07343

**Published:** 2025-12-24

**Authors:** Sady Roberto Rodriguez, Oscar Romero, Marina Guillén

**Affiliations:** Bioprocess Engineering and Applied Biocatalysis Group, Department of Chemical, Biological and Environmental Engineering, Universitat Autònoma de Barcelona, 08193 Bellaterra, Spain

**Keywords:** CO_2_ reduction, Carbon Capture and Utilization, multienzymatic system, coimmobilization, high-value chemicals, waste valorization

## Abstract

Decarbonizing industry demands a shift from high-energy fossil carbon to more sustainable green processes that valorize CO_2_ as a waste product. In this context, biocatalysis offers a promising approach for integrating Carbon Capture and Utilization (CCU) with the conversion of industrial waste into value-added chemicals. To facilitate the transition from bench-scale experiments to industrial-scale CCU, enzyme immobilization plays a crucial role by enhancing biocatalyst stability, reuse, and overall reaction efficiency. This study explores an industrially relevant multienzyme CCU platform to valorize CO_2_ and glycerol by developing a bifunctional biocatalyst through a one-step sequential purification/coimmobilization strategy using formate dehydrogenase (FDH) and glycerol dehydrogenase (GlyDH) for the coproduction of formate and dihydroxyacetone (DHA), with *in situ* cofactor regeneration. The obtained biocatalyst was optimized for stability and activity, and its performance was first evaluated using pure substrates, as well as under industrially relevant conditions with a crude gas mixture mimicking emissions from iron and steel industry and crude glycerol from biodiesel production. The results demonstrate the feasibility of this system for sustainable CO_2_ conversion, achieving the highest formate concentrations reported to date via enzymatic catalysis, 50.4 ± 0.3 mM (2.3 g L^–1^). Similarly, the valorization of crude glycerol into DHA was achieved, along with glycerol carbonate as a byproduct. The biocatalyst-enabled reaction intensification with significant yields for all products improved stability and reusability over five reaction cycles and reduced inhibition. The successful production of three high-value molecules was achieved through a CCU approach aimed at the valorization of industrial waste.

## Graphical abstract

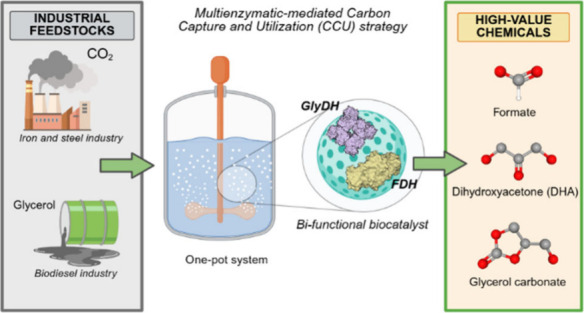

## 1 Introduction

Decarbonizing the industry is a major challenge due to the rapid growth in production and consumption over the last decades. This has resulted in extremely high energy demands and substantial greenhouse gas (GHG) emissions.^1^ These defossilization processes require a significant shift in carbon sources, moving from high-energy fossil carbon to low-energy oxidized carbon such as CO_2_, which is typically released as waste.^2^ Hence, boosting sustainable green chemistry can contribute to reducing or valorizing the carbon dioxide (CO_2_) in industries striving for net-zero emissions.

In carbon-intensive sectors such as iron and steel plants, retrofitting with low-carbon technologies has been shown to potentially reduce CO_2_ emissions by up to 70% by 2050.^3^ This transition can be accomplished through various pathways, including Carbon Capture and Utilization (CCU) technologies, which represent a highly attractive route for using captured CO_2_ in industrial processes, applying the concept of a circular economy. Depending on the process employed—whether chemical, biological, electrochemical, or otherwise—the products generated from CO_2_ (fuels, biodegradable plastics, pharmaceutical precursors, among others) can substitute compounds harmful to the environment, while also creating novel applications and economic opportunities, thereby promoting a circular bioeconomy.^4^ Despite considerable advancements in CCU technologies, significant technical and economic hurdles restrict the deployment of over 50% of these innovations to laboratory settings, often lacking representative industrial environmental conditions. Consequently, only approximately 14% of CCU technologies are currently deemed commercially mature and viable for industrial application.^5^

In the pursuit of greener solutions for CO_2_ mitigation, biocatalysis has emerged as a key alternative for implementing sustainable chemical production. Biocatalysis, in its many manifestations, has the potential to be considered a Green and Sustainable approach, as it aligns with 10 of the 12 principles of green chemistry.^6^ In this context, enzymes are exceptional biocatalysts capable of catalyzing highly complex chemical reactions under mild experimental conditions due to their high selectivity and specificity.^7^

Although enzymes have evolved naturally over millions of years to function within biological systems, this evolution has not been optimized for high-scale industrial conditions such as those in chemical reactors. Disadvantages such as instability, inhibition by substrates and products, and a preference for natural substrates and physiological conditions have somewhat limited their implementation in industrial processes.^8^ Nevertheless, due to advancements in protein engineering, it is now possible to enhance these enzyme properties by leveraging tools such as molecular biology, immobilization and postimmobilization techniques, and innovations in reaction and reactor engineering.^9^

Enzyme immobilization offers an effective strategy to enhance enzyme-mediated CCU at an industrial scale by enabling their reuse, enhancing their stability under challenging reaction conditions and increasing biocatalyst yields.^10^ In CCU technologies, immobilization of enzymes has been shown to significantly reduce the process costs by up to 50%.^11^ Therefore, as a powerful tool for industrial implementation, immobilization has been widely explored in CO_2_ capture technologies as well as in conversion processes.^12^

A wide range of organic, inorganic, and hybrid carriers with excellent physicochemical and rheological properties are available for enzyme immobilization. However, they can present challenges such as enzyme desorption, diffusional limitations reducing activity, high carrier costs, and the need for large amounts of purified enzyme, increasing the biocatalyst’s final cost.^13^ Regarding the immobilization method, affinity interactions between engineered protein tags, such as cellulose-binding domains, chitin-binding domains, peptide tags, and polyhistidine tags (His-tags), which bind strongly to chelated metals are widely reported. These methods facilitate the development of one-step purification-immobilization processes cost, which aim to reduce the purification cost that can represent 50–80% of the total manufacturing cost.^14^ His-tags are particularly valued for maintaining high enzymatic activity, enhancing stability, and minimally affecting protein solubility, structure, or properties.^15^ Thus, selecting an optimal carrier and immobilization method is crucial for maximizing the catalytic efficiency, especially under intensive CO_2_ gas conversion conditions.

In terms of the technical, economic, and environmental viability of CCU technologies, biocatalytic efficiency plays a key role in optimizing sustainable processes, reducing costs, and adapting to industrial conditions.^16^ To approach this scenario, coimmobilizing multiple enzymes on a single support is an effective strategy for deploying multienzyme cascades for CO_2_ conversion. Although coimmobilization can present certain challenges such as suboptimal conditions for one of the enzymes, nonuniform distribution, or cross-inactivation between enzymes, it has also been shown to offer significant advantages. When coimmobilized biocatalysts are used in multienzyme systems, they can improve the efficiency of cascade reactions, increase operational stability, and reduce the accumulation of intermediate products. In addition, there has been a growing interest in multienzymatic platforms for the biotransformation of C1 compounds.^17^ In this context, similar operational conditions of enzymes can be leveraged to integrate multiple enzymatic steps on a single immobilization carrier within a continuous flow system. This strategy promotes more sustainable, cost-effective, and environmentally friendly processes while enabling the production of more complex organic molecules than when using enzymes individually.

In this study, we present a CCU strategy coupled with a multienzyme system using two coimmobilized enzymes for the coproduction of formate, from CO_2_ reduction by formate dehydrogenase (FDH), and dihydroxyacetone (DHA), produced by glycerol oxidation via glycerol dehydrogenase (GlyDH) as a recycling enzyme for cofactor *in situ* regeneration, thus using a bifunctional biocatalyst. The system was successfully tested under an industrially relevant environment using a crude gas mixture mimicking blast furnace off-gas composition from the iron and steel industry and crude glycerol from biodiesel production, demonstrating its feasibility for developing more sustainable CO_2_ valorization bioprocesses.

## 2 Experimental Section

### 2.1 Materials

All reagents were purchased from Sigma-Aldrich (St. Louis, MO, USA) and PanReac Quimica S.L.U. (Barcelona, Spain). The cofactors NADH and NAD^+^ were purchased from GERBU Biotechnik GmbH (Heidelberg, Germany). High-density chelating agarose 6BCL and ReliZyme EP403S were purchased from Agarose Bead Technologies (Madrid, Spain) and Resindion S.r.l. (Binasco, Italy), respectively. EziG Coral and Purolite resins were kindly provided by EnginZyme AB (Stockholm, Sweeden) and Purolite Life Sciences (Pennsylvania, USA), respectively. Alexa Fluor 488 and 610X dyes as well as an Antibody Conjugate Purification kit were purchased from Thermo Fisher Scientific (Massachusetts, USA). All samples and buffers were prepared in Milli Q water (18.2 MΩ·cm, Merck Millipore, USA). Two gas mixtures were used, a pure CO_2_ mixture (24% CO_2_ and 76% N_2_), and a crude CO_2_ mixture (24.5% CO_2_, 46.6% N_2_, 23.9% CO, 1.2% O_2_, and 3.8% H_2_) that mimics the composition of real blast furnace off-gases from iron and steel industry;^18^ both were obtained from Carburos Metallicos (Barcelona, Spain). The crude glycerol (glycerol 64% v/v; full composition in Supporting Information) was kindly provided by ecoMotion Biodiesel S.A. (Barcelona, Spain). Formate dehydrogenase (EC 1.17.1.9) and glycerol dehydrogenase (EC 1.1.1.6) enzymes were produced and purified by the research group according to the procedures found in the SI. Specific activities in cell lysates were 0.79 and 0.85 U mg^–1^ for FDH and GlyDH, respectively.

### 2.2 Enzyme Activity Assays

FDH and GlyDH activities were measured by reduction of NAD^+^ to NADH, driven by formate and glycerol oxidation to CO_2_ and DHA, respectively. For FDH, 100 mM phosphate buffer (pH 7.5), 50 mM sodium formate, and 1.67 mM NAD^+^ was used, while for GlyDH, 100 mM glycerol in 100 mM phosphate buffer (pH 7.0) and 2.5 mM NAD^+^. NADH production was monitored at 340 nm using a Cary 50 Bio spectrophotometer at 30 °C. One unit corresponds to 1 μmol NADH formed per minute.

### 2.3 FDH Immobilization on Different Carriers

FDH immobilization was evaluated on four different IMAC carriers: Ni^2+^-Agarose, EziG Coral, Ni^2+^-ReliZyme, and Ni^2+^-Purolite. Agarose, ReliZyme, and Purolite were previously functionalized as detailed in protocol 1.5 in the SI. EziG Coral carrier was used without modification. Immobilized biocatalysts were prepared by mixing 1 g of carrier with 9 mL of purified enzyme solution in 100 mM phosphate buffer + 100 mM NaCl (pH 7.5) at 4 °C and with a total protein loading of 10 mg g^–1^ (25 U g^–1^). After 60 min, the biocatalysts were filtered, washed, and stored at 4 °C. The following equations were used:

1 $$\mathrm{Immobilization yield}\;\left(\%\right)=\frac{\mathrm{Offered activity}\;\left(U\right)-\mathrm{Supernatant activity}\;\left(U\right)}{\mathrm{Offered activity}\;\left(U\right)}\times 100$$

2 $$\mathrm{Retained activity}\;\left(\%\right)=\frac{\mathrm{Suspension activity}\;\left(U\right)-\mathrm{Supernatant activity}\;\left(U\right)}{\mathrm{Offered activity}\;\left(U\right)-\mathrm{Supernatant activity}\;\left(U\right)}\times 100$$

3 $$\mathrm{Recovery activity}\;\left(\%\right)=\frac{\mathrm{Biocatalyst activity}\;\left(U/g\right)\times \mathrm{Mass of biocatalyst}\;\left(g\right)}{\mathrm{Offered activity}\;\left(U\right)}\times 100$$

4 $$\mathrm{Bound proteins}\;\left(\mathrm{mg}/g\right)=\frac{\mathrm{Offered protein}\;\left(\mathrm{mg}\right)-\mathrm{Supernatant protein}\;\left(\mathrm{mg}\right))}{\mathrm{Mass of carrier}\;\left(g\right)}$$

where *offered activity* is the initial activity in the blank solution (enzyme dissolved in the immobilization buffer), *suspension activity* is the catalytic activity assessed in the solution containing the suspended carrier and the supernatant, *supernatant activity* is the catalytic activity in the supernatant obtained after the suspended carrier was removed by centrifugation, *biocatalyst activity* is the catalytic activity of the carrier obtained at the end of the immobilization, expressed per activity unit per mass of the carrier, *offered protein* is the initial amount of protein in the blank solution, and *supernatant protein* is the amount of protein in the supernatant obtained after removing the resuspended carrier.

### 2.4 Formate Synthesis by Immobilized FDH

Formate synthesis was conducted in a CO_2_-saturated medium (100 mM phosphate buffer pH 7.5) from a pure gas mixture 24% CO_2_ in nitrogen (324 mg CO_2_ L^–1^ equivalent to 7.4 mM) with 10 mM NADH and 0.2 g of biocatalyst in a final volume of 10 mL. Reaction conditions were temperature at 30 °C, 1150 rpm and for 30 h. FDH activity at time zero of the reaction was considered 100% of its operational stability.

### 2.5 Enzymes Coimmobilization

GlyDH and FDH enzymes from cell lysate and purified forms were coimmobilized onto Ni^2+^-ReliZyme carrier at a 1:10 carrier-to-volume ratio. For simultaneous immobilization, both enzymes were prepared in 100 mM phosphate buffer with 100 mM NaCl (pH 7.5) and mixed with the carrier. After complete adsorption, glutaraldehyde at 0.05% v/v was added to the suspension (GC-GlyDH) for enzyme stabilization and maintained at 4 °C for 60 min. The biocatalyst was filtered and washed with immobilization buffer and two imidazole solutions (20 and 50 mM, with 100 mM NaCl).

For sequential immobilization, GlyDH was first offered to the carrier and coated with glutaraldehyde under the same conditions previously described. After that, the biocatalyst was filtered and washed with the same solutions previously described. Subsequently, FDH prepared in the same buffer was added to the GC-GlyDH immobilized biocatalyst. The biocatalyst was filtered, washed with immobilization buffer and imidazole 20 mM + 100 mM NaCl, and stored at 4 °C. Samples were collected during the process to measure catalytic activity in the blank, suspension, and supernatant as well as protein content.

The amount of each enzyme to be immobilized was calculated using the equations described in protocol 1.7 in the SI, considering the previously optimized GlyDH:FDH ratio with free enzymes,^19^ retained activity (without diffusional limitations), protein concentration, enzymatic activity, and expression level in the cell lysates of each enzyme. The maximum protein loading of the Ni^2+^-ReliZyme was determined following protocol 1.8 in the SI.

### 2.6 Study of Biocatalyst Stability

The stability of the bifunctional biocatalyst was evaluated using individually immobilized and coimmobilized GC-GlyDH and FDH on the Ni^2+^-ReliZyme carrier under nonreactive conditions. The effect of the glutaraldehyde coating on GlyDH stability was also examined. All immobilizations were performed using cell lysates with an enzyme loading of 5 mg g^–1^ carrier. Biocatalysts were incubated in 100 mM phosphate buffer (pH 7.5) at 30 °C and 300 rpm for 20 days, and their catalytic activity was periodically measured. Experiments were carried out in duplicate. The half-life (*t*_1/2_) of each biocatalyst was calculated according to protocol 1.11 in the SI.

### 2.7 Inhibition Study of GlyDH by DHA

The inhibition of GlyDH by DHA was evaluated using the enzyme in three forms: free, immobilized, and coimmobilized with FDH on the Ni^2+^-ReliZyme carrier. The effect of different DHA concentrations (0.1, 0.5, 1, 2.5, 5, 10, 25, 50, and 75 mM) on the activity of GlyDH was evaluated. The half maximal inhibitory concentration (IC_50_) was calculated according to protocol 1.12 in the SI.

### 2.8 Multienzymatic Coproduction of Formate and DHA in a Stirred-Tank Reactor

The multienzymatic coproduction of formate and DHA was carried out in a stirred-tank reactor by a bifunctional biocatalyst, with *in situ* NADH regeneration and continuous CO_2_ supplementation. The reaction medium consisted of 100 mM phosphate buffer (pH 7.5), 1 mM NADH, 100 mM glycerol, and continuous gas bubbling at 1 vvm from a pure gas mixture containing 24% CO_2_ in nitrogen. For the reaction under industrially relevant conditions, a crude CO_2_ gas mixture mimicking iron and steel industry emissions (24.5% CO_2_)^18^ and a crude glycerol from biodiesel production were used (full composition in the SI, Table S1). A total of 20 g of bifunctional biocatalyst were resuspended in a final volume of 200 mL. Experiments were conducted by duplicate at 30 °C with constant stirring at 300 rpm. Samples were collected periodically to measure substrates, products, and enzyme activities (procedures detailed in the SI).

### 2.9 Reusability of the Bifunctional Immobilized Biocatalyst

The reusability of the bifunctional biocatalysts was evaluated by several consecutive reaction cycles. Biocatalyst losses were compensated by reducing the reaction volume to maintain a constant concentration of 100 g L^–1^ and performing the reaction at the same time. The biocatalyst loss after five cycles was less than 7.5%. After each cycle, the biocatalyst was washed with 100 mM phosphate buffer (pH 7.5) using the same volume as the reaction. The product concentration in cycle 1 was defined as 100% yield. The following equations were used:

$$\mathrm{Space Time Yield}\;\left(\mathrm{STY}\right)=\frac{\mathrm{Product mass}\;\left(\mathrm{mg}\right)}{\mathrm{Reaction volume}\;\left(L\right)\times \mathrm{Time}\;\left(h\right)}$$ 5

$$\mathrm{Cumulated STY}\;\left(\mathrm{cSTY}\right)=\frac{\Sigma \left[\mathrm{Product mass}\;\left(\mathrm{mg}\right)\;\mathrm{total cycles}\right]}{\mathrm{Reaction volume}\;\left(L\right)\times \Sigma \left[\mathrm{Time}\;\left(h\right)\;\mathrm{total cycles}\right]}$$ 6

7 $$\mathrm{Catalyst yield}=\frac{\mathrm{Product mass}\;\left(\mathrm{mg}\right)}{\mathrm{Catalyst}\;\left(g\right)\;\mathrm{or protein}\;\left(\mathrm{mg}\right)}$$

8 $$\mathrm{Cumulative catalyst yield}\;\left(\mathrm{cCY}\right)=\frac{\Sigma \left[\mathrm{Product mass}\;\left(\mathrm{mg}\right)\;\mathrm{total cycles}\right]}{\mathrm{Catalyst}\;\left(g\right)\;\mathrm{first cycle}}$$

### 2.10 Assessment of the Environmental Efficiency (E-Factor)

The environmental impact of this multienzymatic CO_2_ reduction process was evaluated through the determination of the environmental factor (E-factor), calculated according to the equation proposed by Sheldon^20^ for the three products obtained in the first reaction cycle:

9 $$E\text{‐}\mathrm{factor}=\frac{\mathrm{mass of waste generated}\;\left(\mathrm{kg}\right)}{\mathrm{mass of product obtained}\;\left(\mathrm{kg}\right)}$$

where

Mass of waste=(Mass of
reaction media−mass of biocatalyst−mass of products)

Mass of products=(Mass of
formate+DHA+Glycerol carbonate)

## 3 Results and Discussion

### 3.1 Selection of Immobilization Carrier

First, the selection of an appropriate immobilization carrier for FDH was guided by evaluating the immobilization yield, recovered activity, operational stability, and efficiency of formate production from CO_2_. For that, four different carriers were studied: Ni^2+^-Agarose, EziG Coral, Ni^2+^-ReliZyme, and Ni^2+^-Purolite. Table 1 summarizes the key findings, with additional parameters detailed in the SI (Table S2).

**Table 1:** Evaluation of Different Carriers for FDH Immobilization Based on Recovered Activity, Immobilization Yield, Operational Stability, and Formate Synthesis

	immobilization parameters		reaction parameters	
carrier	recovery activity (%)	immobilization yield (%)^a^	operational stability (%)^b^	formate synthesis (mM)
Ni^2+^-Agarose	94.2 ± 0.1	100 ± 0.2	89 ± 0.2	0.12 ± 0.02
EziG Coral	71.5 ± 0.4	83.9 ± 0.2	40.1 ± 0.3	0
Ni^2+^-ReliZyme	94.5 ± 0.1	100 ± 0.2	91.3 ± 0.2	1.5 ± 0.1
Ni^2+^-Purolite	87.7 ± 0.3	100 ± 0.1	67.7 ± 0.4	0

a Immobilization yield in terms of bound protein.

b Residual activity under reaction conditions after 30 h.

As shown, FDH immobilization on Ni^2+^-Agarose and Ni^2+^-ReliZyme showed the best performance in terms of recovered activity (94.2 ± 0.1% and 94.5 ± 0.1%) and operational stability (89 ± 0.2% and 91.3 ± 0.2%). Moreover, formate synthesis was detected only on these two carriers, with Ni^2+^-ReliZyme yielding the highest formate production (1.5 ± 0.1 mM). The Ni^2+^-Purolite carrier resulted in a slight decrease in the FDH activity recovery (87.7 ± 0.3%). In contrast, the EziG Coral carrier exhibited the lowest recovery (71.5 ± 0.4%) and yield of 83.9 ± 0.2% based on protein content. In these two carriers, significant FDH inactivation under reactive conditions was observed, with the EziG Coral carrier exhibiting the highest inactivation (40.1 ± 0.3%). After 30 h of reaction, no formate formation was observed on either of these carriers. Due to the poor performance observed in the evaluated parameters, EziG Coral and Ni^2+^-Purolite carriers were discarded.

Agarose is a widely used support for enzyme immobilization due to its hydrophilic nature, high thermal resistance, and well-defined particle size. However, its fragility under mechanical stress, high cost, and diffusional limitations at high protein loading restrict its industrial applicability.^21^ In contrast, ReliZyme offers superior mechanical stability, microbial resistance, and minimal swelling in aqueous environments, making it a more robust alternative for both laboratory and industrial use. Its larger surface area allows for higher protein immobilization, which may explain the improved formate synthesis observed with this carrier. Therefore, based on these results, Ni^2+^-ReliZyme was selected as the optimal carrier for high-intensity conditions due to its mechanical stability, suitable pore size (40–60 nm) for gas diffusion, and cost-effectiveness compared to agarose. Its use in large-scale enzyme immobilization is well documented.^22^

### 3.2 Development of a Bifunctional Biocatalyst from Cell Lysates by One-Step Purification/Coimmobilization

Following the selection of Ni^2+^-ReliZyme as the immobilization carrier, the coimmobilization of both enzymes was investigated for the obtaining of a bifunctional biocatalyst. Regarding GlyDH, it has been reported that multimeric enzymes like this are highly susceptible to subunit dissociation and destabilization upon immobilization.^23^ Thus, postimmobilization modifications are crucial to improve stability and extend the enzyme’s operational lifetime. A study showed that cross-linking with 0.05% (v/v) glutaraldehyde creates covalent bonds with amino groups on the glutaraldehyde-coated GlyDH (GC-GlyDH), stabilizing its structure. This concentration could prevent subunit dissociation and retain up to 50% activity for over 25 days. However, glutaraldehyde addition can significantly reduce the enzyme’s expressed activity due to extensive chemical modifications.^24^

When coimmobilizing enzymes, different strategies based on the order can be approached. In this work, coimmobilization was carried out simultaneously by offering a mixture of both enzymes at the same time and sequentially by first offering GlyDH and then FDH (Figure 1). Both experiments were carried out at a low enzyme load (2 mg of protein g^–1^ carrier). In both strategies, GC-GlyDH loses around 50% of its initial activity due to glutaraldehyde coating, as previously discussed (Figures 1A and 1B). Regarding FDH, a significant loss of activity (95.9 ± 0.3%) was observed in the simultaneous coimmobilization (Figure 1A). This is attributable to the glutaraldehyde, which may induce structural modifications and a substantial loss of catalytic activity in some other proteins.^24^ Due to this, in the sequential immobilization, FDH was immobilized after the coating and washing of the GC-GlyDH. As a result, FDH successfully retained 95.4 ± 1.3% of its activity due to the complete removal of glutaraldehyde prior to its immobilization (Figure 1B).

**Figure 1:**
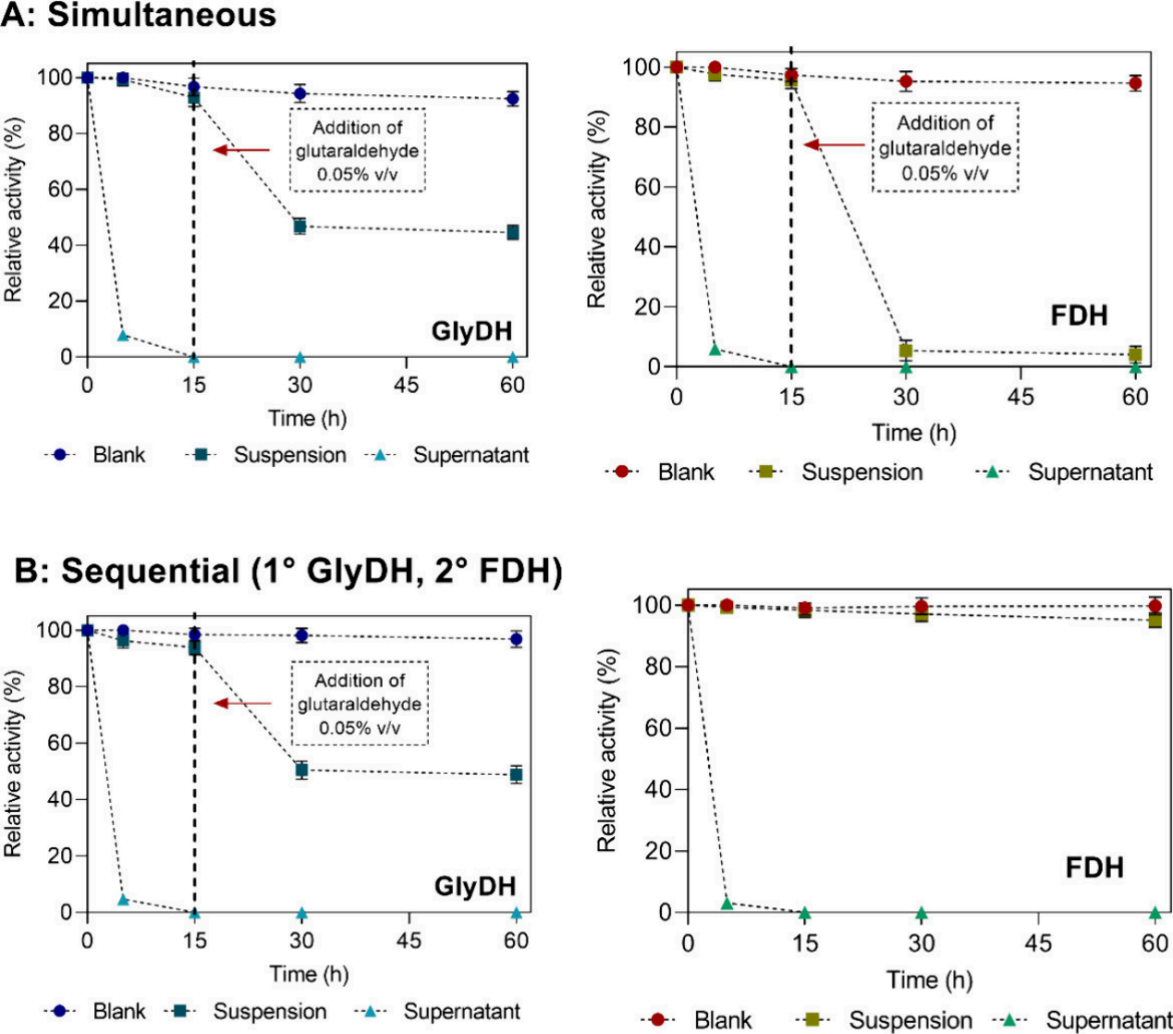
Simultaneous and sequential coimmobilization of purified GlyDH and FDH on Ni^2+^-ReliZyme with a protein load of 2 mg g^–1^ carrier. The relative activity was calculated by considering the activity offered for each enzyme as 100%. (A) Simultaneous coimmobilization. (B) Sequential coimmobilization.

Although simultaneous coimmobilization is simpler, it can hinder control over enzyme ratios and cause competition for carrier binding sites. In contrast, sequential coimmobilization, though more time-consuming and prone to pore blockage, allows better control of enzyme loading, orientation, and stepwise optimization.^25^ As a result, sequential coimmobilization was selected as the optimal strategy to preserve GC-GlyDH stability through cross-linking while preventing FDH inactivation by glutaraldehyde.

To reduce the high costs associated with protein purification, which can represent up to 80% of the total production cost,^14^ coimmobilization of GlyDH and FDH from cell lysates was carried out at a low protein load (2 mg g^–1^ carrier). Table S3 in the SI summarizes the parameters evaluated in the coimmobilization process, comparing purified enzymes with cell lysates. Coimmobilization using cell lysates was successfully carried out, indicating the high specificity of the enzyme His-tag for nickel ions on the carrier, which facilitated the one-step purification/coimmobilization process of these two enzymes. It has been reported that affinity-based immobilization (with His-tag proteins) enables selective and oriented binding, often preserving activity and achieving high purity in processes that simultaneously purify and immobilize recombinant proteins. In addition, its reversibility allows carrier reuse but can reduce binding stability; therefore, it is sometimes combined with covalent binding and cross-linking strategies to develop more robust heterofunctional carriers. Despite these limitations, affinity-immobilized enzymes have numerous applications in the biotechnology, food, and protein purification industries.^26^ As a result, the recovered activities in the bifunctional biocatalyst prepared from cell lysates were 43.9 ± 0.6% for GC-GlyDH and 94.8 ± 0.8% for FDH.

Additionally, the stability of the individual immobilized enzymes and the coimmobilized biocatalyst was analyzed (Figure 2). The results demonstrated that glutaraldehyde coating markedly increased the half-life (*t*_1/2_) of immobilized GlyDH by 1.9-fold, extending it from 6.5 to 12.5 days. Furthermore, coimmobilization led to an even greater enhancement in GlyDH stability, with a *t*_1/2_ of 13.7 days, representing a 1.1-fold improvement compared to the individually immobilized GC-GlyDH (Figure 2A). Thus, despite the loss of catalytic activity caused by glutaraldehyde-induced structural changes, GC-GlyDH showed enhanced stability, mainly due to covalent bond formation that helped preserve the enzyme’s structural integrity and stability over time.^24^ An improvement in stability was also observed for FDH, where coimmobilization extended its *t*_1/2_ from 12.8 to 15.8 days, a 1.2-fold improvement (Figure 2B). After 20 days of incubation, the residual activity in the bifunctional biocatalyst was 22.1 ± 1.2% and 28.3 ± 0.8% for GC-GlyDH and FDH, respectively, highlighting the long-term stability achieved. As a result, coimmobilization not only potentially increases functional synergy by creating a favorable microenvironment for the enzymes but also enhances their stability through protective mechanisms that prevent denaturation and inactivation of the enzymes involved.^27^

**Figure 2:**
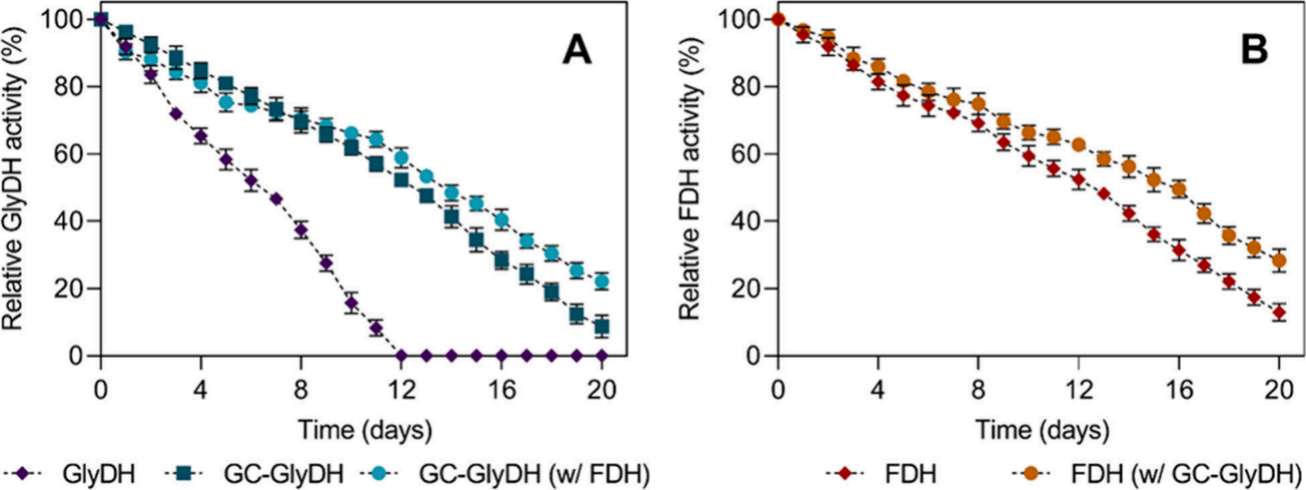
Stability study of GlyDH and FDH immobilized/coimmobilized on the Ni^2+^-ReliZyme carrier under nonreactive conditions. Immobilizations were carried out with an enzyme loading of 5 mg g^–1^ carrier using cell lysates: (A) GC-GlyDH and (B) FDH.

After optimizing the coimmobilization of GC-GlyDH and FDH at low enzyme loading (2 mg g^–1^) directly from cell lysates, higher protein loadings were tested to determine the carrier’s maximum capacity, enabling the multienzymatic reaction at a larger scale. In order to avoid unspecific interactions with other proteins, each biocatalyst was washed with low concentrations of imidazole and sodium chloride to remove nonspecific proteins, following the scheme in Figure S3. In addition, an SDS-PAGE analysis of the samples collected at each washing stage is shown in Figure S4, along with a brief discussion. Tables S4 and S5 in the SI show the results of individual immobilizations of GC-GlyDH and FDH at different protein loadings. The Ni^2+^-ReliZyme carrier showed a high surface area suitable for effective attachment of high amounts of proteins, reaching immobilization yields of 98.5 ± 0.1% for GC-GlyDH and 100 ± 0.3% for FDH when about 50 mg of each enzyme was bound individually. Due to their large pores and high surface functionality, ReliZyme carriers are capable of immobilizing large amounts of proteins, as previously reported for other enzymes.^28^ Regarding recovery activity, significant diffusional limitations were observed upon GC-GlyDH immobilization, resulting in reduced activity, which is further exacerbated by the cross-linking effect of glutaraldehyde. Beyond ∼25 mg (87.5 ± 0.4 U g^–1^ GC-GlyDH offered), the observed activity plateaued at 13.6 ± 0.3 U g^–1^, indicating carrier surface saturation (Figure S5 in the SI). For FDH, carrier saturation appeared at ∼50 mg (79.7 ± 0.6 U g^–1^ FDH observed), suggesting a more favorable enzyme arrangement for higher activity recovery (Figure S5). Some studies have shown that the ReliZyme’s particle size and high porosity can significantly affect mass transfer, causing diffusional limitations of substrates and products to enzyme active sites. Nonetheless, ReliZyme carriers have been shown to be generally robust, providing rigidity and stability even at high concentrations of immobilized enzymes.^29^

Based on these results, the bifunctional biocatalyst was prepared with a high load of both enzymes, as shown in Table 2. The immobilization kinetics is shown in Figure S6. As a result, the final activities observed in the biocatalyst were 11.3 ± 0.2 and 8.7 ± 0.5 U g^–1^ for GC-GlyDH and FDH, respectively, in contrast with the theoretical activities of 91.8 ± 2.1 and 9.8 ± 0.4  U  g^–1^, respectively. Consequently, ReliZyme carrier provides an optimal platform for attaching high protein loads, enabling the multienzymatic reaction to be carried out on a larger scale.

**Table 2:** Summary of the Parameters Evaluated in the Sequential Coimmobilization of GC-GlyDH and FDH (from Cell Lysates) onto Ni^2+^-ReliZyme^a^

parameters	GC-GlyDH	FDH
offered activity (U g^–1^)	209.5 ± 2.5	10.3 ± 1.5
theoretical biocatalyst activity (U g^–1^)	91.8 ± 2.1	9.8 ± 0.4
observed biocatalyst activity (U g^–1^)	11.3 ± 0.2	8.7 ± 0.5
yield–activity (%)	100 ± 0.5	100 ± 0.2
retained activity (%)	14.0 ± 0.3	88.2 ± 2.5
recovery activity (%)	10.8 ± 0.4^a^/4.9 ± 0.4	84.0 ± 0.3
offered proteins (mg)	3620.4 ± 1.9	378.2 ± 2.3
bounded proteins (mg g^–1^)	40.8 ± 1.2	9.8 ± 0.6
yield–proteins (%)	22.6 ± 1.3	51.6 ± 1.8
purification factor	4.4	1.9

a GC-GlyDH: GlyDH coated with glutaraldehyde.

b GC-GlyDH recovery activity before FDH addition.

Finally, the enzyme distribution in the carrier’s pore was characterized by confocal microscopy with fluorophore-labeled GlyDH and FDH, prepared with the same protein load as the biocatalyst used for the reaction. As shown in Figure S7A, the Ni^2+^-ReliZyme carrier has a spherical shape with a diameter of at least 50 μm and displays a slight translucent appearance with no fluorescence at 490 or 603 nm. Regarding the individual FDH and GlyDH immobilization, the observed fluorescence corresponds to enzymes immobilized within the pores located nearest to the resin surface, although some enzyme may also be present within the inner pores of the particle (Figures S7B and S7C). The homogeneous distribution of both enzymes on the surface suggests that they have a similar affinity for the carrier. Finally, in the bifunctional biocatalyst, an overlap of the green and red signals is observed, indicating colocalization of both enzymes at the same sites (Figure S7D). A homogeneous distribution of both enzymes on the carrier enhances catalytic performance by increasing cofactor-enzyme encounters and minimizing diffusion limitations. Close enzyme proximity in cascades may improve reaction efficiency, while preventing enzyme clustering reduces dead zones and maximizes surface activity.^30^

### 3.3 Intensification of the Coproduction of Formate and DHA by a Bifunctional Biocatalyst

Following the development of the bifunctional biocatalyst, the intensification of the CO_2_ reduction reaction was implemented. This involved the multienzymatic coproduction of formate and DHA, coupled with *in situ* NADH regeneration and continuous CO_2_ supplementation in a 200 mL stirred-tank reactor. Since volumetric gas flow rates affects gas–liquid mass transfer, 1 vvm from a 24% CO_2_ gas mixture was selected to carry out the experiments, which is considered a feasible and standard value of gas flow rate per reactor volume,^31^ to facilitate the progression of the reaction. A scheme of the experimental setup is shown in the Figure S2.

Figure 3A shows the time course of the reaction with pure substrates (pure gas mixture with 24% CO_2_, and pure glycerol). As shown, formate detection becomes evident after approximately 12 h of reaction, a behavior also observed in the formate and DHA coproduction with free enzymes.^19^ After that, the production kinetics follow a more linear trend, reaching a final concentration of 50.4 ± 0.3 mM (2.3 g L^–1^) after 80 h of reaction, which represents the highest concentrations reported so far in the literature for this molecule via enzymatic synthesis. Compared to other multienzymatic platforms and cofactor regeneration systems, the formate concentration achieved in this study significantly outperforms existing reports.^32,33^ This milestone marks a significant advancement in immobilized biocatalysis, achieving an approximately 8-fold enhancement compared to the free enzyme system.^19^

**Figure 3:**
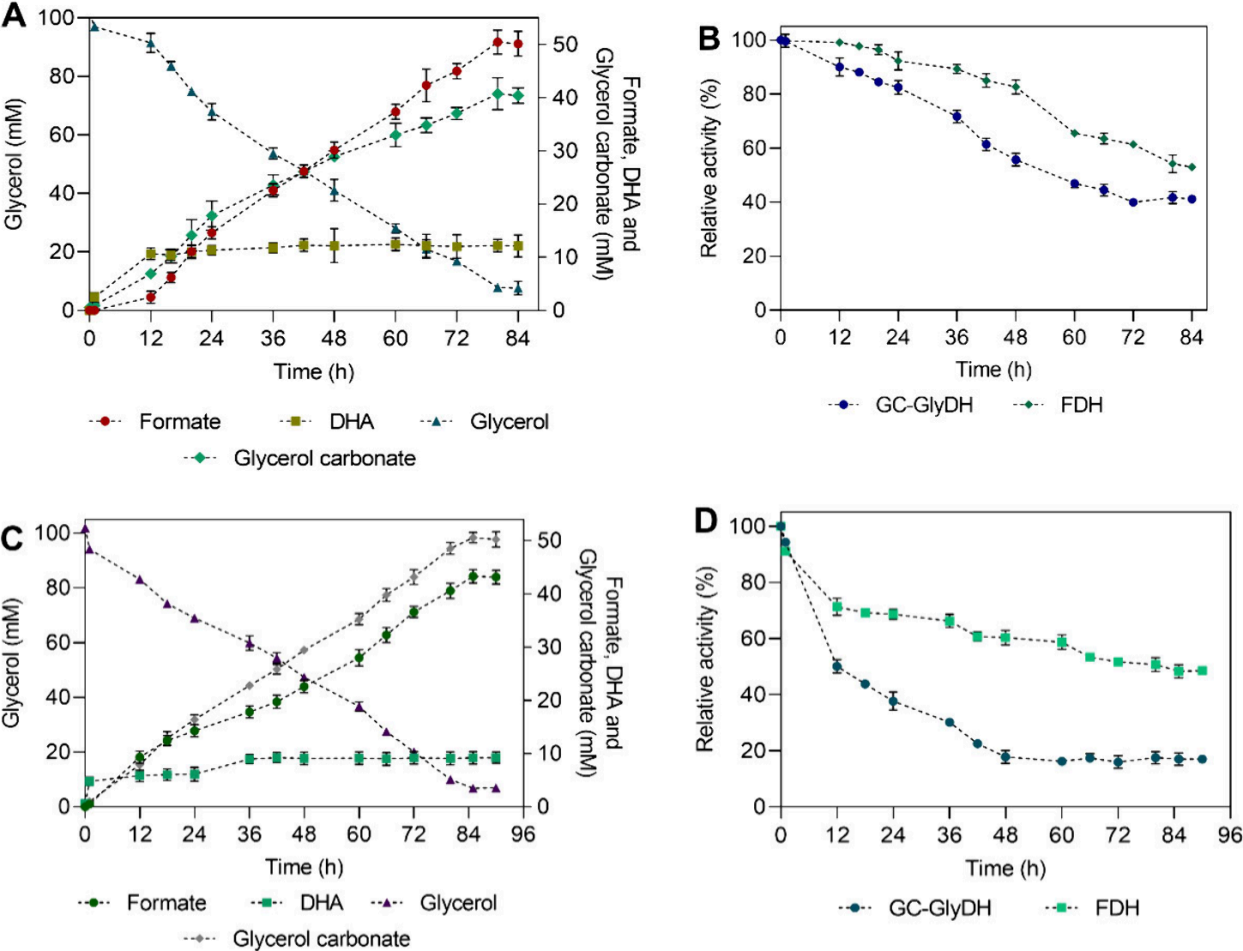
Multienzymatic coproduction of formate and DHA with NADH *in situ* regeneration and continuous CO_2_ supplementation. (A and B) Time course and biocatalyst operational stability of the reaction with a pure gas mixture of 24% CO_2_ + pure glycerol. (C and D) Time course and biocatalyst operational stability of the reaction under relevant industrial conditions (crude CO_2_ gas mixture mimicking off-gases from iron and steel industry + crude glycerol). 100% residual activity was defined as the activity at the reaction’s initial time.

Concerning DHA, it was detected from the reaction’s onset (Figure 3A), with a final concentration of only 12.2 ± 0.4 mM (equivalent to 1.1 g L^–1^), suggesting possible adsorption onto biocatalyst. To explore this, DHA adsorption isotherms were determined for both the carrier (Ni^2+^-ReliZyme) and the bifunctional biocatalyst. The results, detailed in Table S6 and Figure S8, clearly show a strong affinity of DHA for both adsorbents, with maximum adsorption capacities of 62.6 ± 0.3 and 140.8 ± 0.5 mg of DHA g^–1^, respectively. This behavior is evident after 12 h, maintaining a stable concentration throughout the reaction. DHA may preferentially adsorb onto the biocatalyst due to the increased availability of amino groups from the enzymes, which interact favorably with DHA’s keto-triose structure and its potential to undergo Maillard reactions.^34^ While Ni^2+^-ReliZyme offers a general environment for molecule retention due to its physicochemical properties, the enzymes’ amino groups exhibit a stronger affinity for DHA, consistent with literature.^35^

On the other hand, a glycerol conversion of 92.3 ± 0.9% was achieved, corresponding to a final concentration of 7.9 ± 1.2 mM. Notably, glycerol carbonate (GC) was identified as a byproduct, formed via direct carboxylation of glycerol with CO_2_, releasing water^36^ (reaction mechanism in Figure S9). While the authors previously observed this byproduct in the reaction with free enzymes,^19^ the concentration obtained here (40.7 ± 0.2 mM, equivalent to 4.8 g L^–1^ after 80 h) was significantly higher, accounting for the consumption of the remaining glycerol. GC is also a high-value molecule from the valorization of bioglycerol, and its production has been extensively reported with metal catalyst (such as Zn^2+^, Pt^2+^, Ni^2+^, and Al^3+^ among others) and from substrates such as ethylene carbonate, dimethyl carbonate, diethyl carbonate, and dibutyl carbonate.^37^ Therefore, the potential to produce GC directly from CO_2_ offers a considerably more sustainable and economically attractive route.

To understand the synthesis of GC under these conditions, a reaction of 100 mM glycerol with the pure gas mixture 24% CO_2_ in the presence of zinc sulfate and the immobilization carrier without enzymes (Ni^2+^-ReliZyme) was performed, achieving 5.8 ± 0.2 and 25.7 ± 0.1 mM, respectively. Hence, the nickel on the carrier and the zinc in the metalloenzyme GlyDH may catalyze the synthesis of this byproduct. Nickel is commonly used in GC synthesis, however, with carbonates organic and salts as substrates.^38^ On the other hand, zinc has also been reported as a catalyst in this synthesis.^39^ This metal stabilizes the GlyDH’s structure, ensuring proper conformation and catalytic activity. It also activates glycerol by stabilizing negative charges in the reaction intermediate, promoting deprotonation to the glyceroxide anion, which is key for GC synthesis.^40^ Thus, the production of GC may be catalyzed by these two metals under the reaction conditions.

To assess the feasibility of the bifunctional biocatalyst, its operational stability was examined (Figure 3B). The data show a gradual inactivation of both enzymes over time, with GC-GlyDH and FDH retaining 41.7 ± 0.2% and 54.2 ± 0.2% of their initial activities, respectively, by the end of the reaction. In general, continuous gas bubbling may be a major inactivator for both free enzymes as it destabilizes them at the gas–liquid interface. After 24 h of continuous CO_2_ bubbling at 10 mL min^–1^, free FDH was inactivated by 63 ± 0.1%.^19^ These results highlight the crucial role of immobilization in enhancing the enzyme operational stability in this system.

On the other hand, despite the widely reported inhibition of GlyDH by DHA from low concentrations (0.4–0.54 mM),^41^ GC-GlyDH remained active even at higher DHA concentrations. As shown in Figure S10, immobilized GC-GlyDH retains 36 ± 1.9% of its activity at the highest DHA concentration tested (75 mM), while coimmobilization with FDH further enhances activity, reaching 44.2 ± 2.5%. According to Rocha-Martin et al., immobilizing GlyDH could potentially reduce DHA inhibition, increasing activity by up to 3.7 times.^42^ In this work, the IC_50_ increased from 0.33 mM DHA with free GlyDH to 16.8 mM DHA when GC-GlyDH was coimmobilized with FDH, representing a 51-fold increase in tolerance to inhibition. For immobilized GC-GlyDH alone, the IC_50_ for DHA was 1.06 mM, representing a 3.2-fold increase compared with the free enzyme. Therefore, enzyme immobilization is not only an effective strategy for enhancing stability and prolonging lifespan but may also induces slight conformational changes in the active site, reducing inhibition by product. Additionally, protein–protein interactions may stabilize GlyDH or modify its kinetics, reducing inhibition sensitivity.^43^ Hence, the successful coproduction of three high-value molecules was achieved at outstanding concentrations by an efficient bifunctional biocatalyst.

To address the limited industrial translation of most CCU technologies, we assessed the multienzymatic system under industrially relevant conditions using a crude CO_2_ gas mixture that mimics blast furnace off-gases from the iron and steel industry (24.5% CO_2_, 46.6% N_2_, 23.9% CO, 1.2% O_2_, and 3.8% H_2_),^18^ along with a crude glycerol from biodiesel production. Figure 3C illustrates the time course of the reaction with the industrial substrates.

The successful production of all three high-value molecules was achieved under these conditions. Formate and glycerol carbonate exhibited relatively linear production kinetics, alongside glycerol consumption, similar to the reaction with free enzymes.^19^ A similar glycerol conversion was achieved in this reaction, 93.4 ± 1.2%. Formate synthesis was 43.3 ± 1.3 mM (equivalent to 2 g L^–1^), which represents a 14.1% lower yield than in the reaction with pure substrates, with a slower reaction rate and maximum yield reached at 85 h. Notably, a higher glycerol carbonate yield, 50.6 ± 0.9 mM (equivalent to 6 g L^–1^), was achieved, due to lower coproduction of formate and DHA, favoring glycerol consumption for its synthesis. Regarding DHA, a low concentration in the reaction medium was obtained, 9.2 ± 0.2 mM (equivalent to 0.8 g L^–1^), indicating a slightly higher adsorption (3% more than with pure substrates). This may be related to the highly heterogeneous composition of both substrates and modifications of the reaction environment, leading to increased DHA adsorption. However, further studies are needed to clarify this phenomenon.

Regarding the bifunctional biocatalyst’s operational stability (Figure 3D), GC-GlyDH showed a rapid loss of activity in the first 48 h, followed by a stabilization of its activity. At the end of the reaction, the residual activity of GC-GlyDH was only 17 ± 2.2% (24.7% less than with pure substrates) and for FDH was 48.3 ± 0.2%, (5.9% less than reaction with pure substrates). This enzyme inactivation may have an impact on the reaction rate and yields of formate and DHA coproduction, likely due to crude substrates composition, which may induce different levels of inactivation, as previously reported for the system using free enzymes.^19^ Gases like carbon monoxide (CO) may cause significant inactivation of oxide-reductive enzymes.^44^ Additionally, the presence of toxic species like methanol and the fat content in crude glycerol, which may contribute to the formation of surface-active species, leading to a loss of enzymatic activity.^45^ However, despite these challenges, formate and GC synthesis from a crude gas mixture simulating emissions from the iron and steel industry was successfully achieved, and the valorization of crude glycerol into DHA and GC was also accomplished. This latter approach allows avoiding the high costs associated with crude glycerol purification while minimizing the environmental impact caused by its disposal through incineration.^46^

### 3.4 Robustness of the Multienzymatic System through Biocatalyst Reusability

To assess the robustness and reusability of the bifunctional biocatalysts in both reactions, five consecutive reaction cycles were performed maintaining the same conditions. Figure 4 displays the yields for each product along with the biocatalyst operational stability across the five cycles. In the reaction with the pure substrates, formate synthesis showed an impressive yield of 70 ± 2.5% after five cycles (400 h of operation). In the second and third cycles, the yield dropped from 100% to 84.6 ± 2.2% and 77.7 ± 2.6%, but the decline was less pronounced in subsequent cycles. This trend may be linked to FDH activity, which showed an 8% decrease in residual activity after the second and third cycles; however, in the later cycles, the decline was minimal, at only 1% (Figure 4B), suggesting a remarkable operational stability. Regarding GC-GlyDH, a similar behavior is observed, with negligible inactivation across all reaction cycles (Figure 4B).

**Figure 4:**
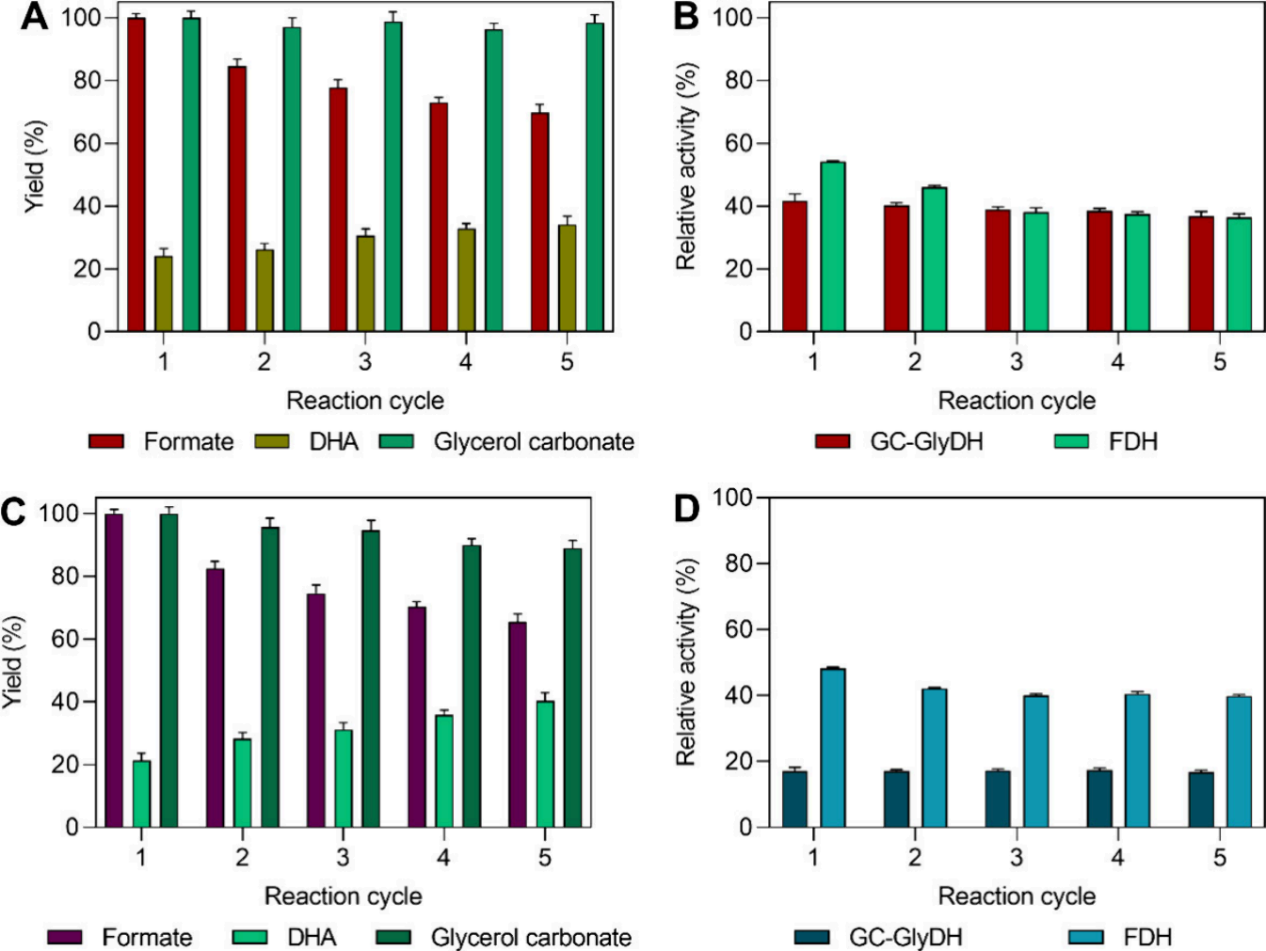
Robustness of the multienzymatic system through biocatalyst reusability over five consecutive reaction cycles. (A and B) Yields and operational stability of the biocatalyst in the reaction with the pure gas mixture of 24% CO_2_ + pure glycerol. (C and D) Yields and operational stability of the biocatalyst under industrially relevant conditions (crude CO_2_ gas mixture mimicking off-gases from iron and steel industry + crude glycerol). Yields and residual activity from the first cycle at the initial reaction time were defined as 100% of the reference values.

For DHA, its concentration in the liquid medium increased with each cycle, reporting 17.3 ± 0.2 mM (equivalent to 1.6 g L^–1^) by the fifth cycle, a 41.8% increase compared to that of cycle 1 (Figure 4A). This suggests that the biocatalyst became progressively saturated with DHA, leading to a reduced adsorption capacity over successive cycles. Table S7 in the SI shows a mass balance of DHA adsorption on the biocatalyst throughout the cycles. As observed, after five cycles, an estimated 2253.3 ± 3.5 mg of DHA was adsorbed onto the biocatalyst, corresponding to a capacity of 116.3 ± 1.8 mg per gram of biocatalyst. This represents 82.6% of the biocatalyst’s maximum adsorption capacity, 140.8 mg of DHA g^–1^ (Table S6), suggesting that further cycles could saturate it, leaving the whole DHA produced in the soluble medium.

Glycerol carbonate production shows a consistent trend across all reaction cycles (Figure 4A), primarily driven by metal catalysis, as previously discussed, suggesting that enzymatic inactivation should not affect its synthesis, and the nickel remains stable throughout the reaction with minimal degradation. Other nickel- and zinc-based catalysts have also been reported to retain their activity and GC productivity over multiple cycles.^47^

In the case of the reaction under relevant industrial conditions, the formate yield was 65.5 ± 2.5% by the fifth cycle, 4.4% less than with pure substrates (Figure 4C). A notable decrease was observed in the second cycle, with the yield dropping from 100% to 82.5 ± 2.2%, however, in subsequent cycles, productivity differences were minor, including for the other two products. This behavior may also be attributed to the operational stability of the enzymes involved (Figure 4D). In cycle 2, FDH exhibited a 6.1% decrease in activity compared with cycle 1, but residual activity stabilized in subsequent cycles. Meanwhile, GC-GlyDH demonstrated remarkable operational stability, despite significant inactivation in the first cycle. Therefore, the biocatalyst exhibited a high operational stability, indicating excellent reusability and robustness under these conditions.

Regarding DHA, its concentration in the liquid medium increased over the reaction cycles, with soluble proportions slightly higher compared to the reaction with pure substrates (6.1% more), likely due to competition with other compounds present in both substrates, which may reduce DHA adsorption (Figure 4C). The mass balance for DHA in this reaction estimated a total adsorption of 1802 ± 2.2 mg, corresponding to 91.9 ± 2.1 mg of DHA per gram of biocatalyst (Table S7). This represents 65.3% of the maximum adsorption capacity of the biocatalyst and 451.3 mg less DHA adsorbed than with pure substrates. These differences from the reaction with pure substrates likely stem from the heterogeneous crude substrate composition, which may affect the adsorption equilibrium and reaction conditions; however, further studies are needed to clarify this behavior.

In the case of glycerol carbonate, its concentration gradually declined from 50.6 ± 0.97 mM in the first cycle to 44.9 ± 0.5 mM in the fifth cycle (Figure 4C), likely due to crude substrate characteristics or continuous catalyst degradation. Metal poisoning is a common degradation mechanism in these type of catalysts, where adsorption of certain substances blocks active sites. For nickel, contact with compounds such as carbon monoxide (CO), which is present in the crude gas mixture, may strongly adsorb on the surface, thereby reducing its activity.^48^ Likewise, although some nickel- and zinc-based catalysts show high glycerol conversion and GC selectivity, repeated cycles can also induce surface modifications that negatively affect GC productivity.^49^

Finally, Figure 5 shows both the cumulative space–time yield (cSTY) and cumulative catalyst yield (cCY) on the five sequential batches carried out. The results demonstrate cumulative STY of 22.1 and 17.6 mg L^–1^ h^–1^ in the formate synthesis for the pure and crude substrates (Figure 5A), representing a significant enhancement of 2.6 and 3.7-fold, respectively, compared to the reaction with free enzymes.^19^ Regarding the catalyst yield, 88.4 and 74.8 mg g^–1^ were achieved (Figure 5B). As shown in Table 3, these formate yields are comparable to or even surpass those reported in enzymatic studies involving a similar number of biocatalyst reuse cycles. Compared with other platforms, such as electrochemical and bioelectrocatalytic systems, this system also achieves formate yields comparable to those reported in some studies.^50,51^ However, in certain cases, electroreduction can deliver substantially higher CO_2_ conversion to formate yield, up to seven times greater than those observed in this study, due to faster reaction kinetics and greater scalability.^52,53^ Nevertheless, these electrochemical systems often suffer from rapid catalyst degradation, limited selectivity, and high energy requirements.

**Figure 5:**
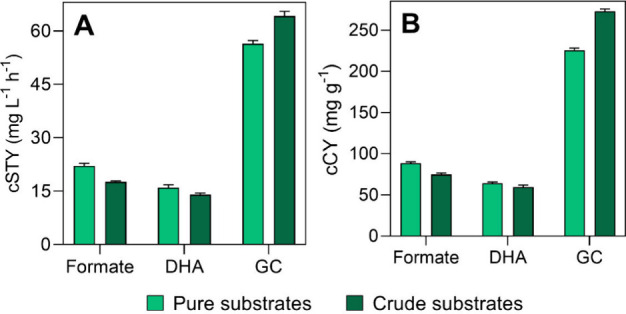
Comparison of the (A) cumulative STY (cSTY) and (B) cumulative catalyst yield (cCY) obtained in the multienzymatic CO_2_ reduction reaction carried out with pure substrates (gas mixture of 24% CO_2_ + glycerol) and the reaction with crude substrates (crude gas mixture from the iron and steel industry + crude glycerol) over five consecutive reaction cycles. STY: space–time yield.

**Table 3:** Literature Reports on the Enzymatic Synthesis of Formate and DHA, as well as Glycerol Carbonate from an Immobilized Biocatalyst

molecule	catalyst	carrier immobilization	system	concentration (mM)	cumulative STY yield (mg L^–1^ h^–1^)	reaction cycles	reference
formate	FDH	carbon felt	CO_2_ reduction at 8 bar	7.6	24.9	4	^51^
formate	FDH	electrospun polystyrene nanofibers (EPSNF)	NADH electrochemical regeneration with a copper foam electrode	0.31	2.7	--	^57^
formate	FDH	Zeolitic Imidazolate Framework-8 (ZIF-8)	multienzymatic system with carbonic anhydrase	0.3	0.9	--	^32^
formate	FDH	hierarchically porous metal organic framework (HP-UiO-66-NH_2_)	electrocatalytic NADH regeneration	1.83	27.0	5	^50^
formate	FDH	mesoporous silica	not NADH regeneration and low recovery activity	0.08	1.8	5	^33^
formate	FDH	metal–organic frameworks (MOFs)	NADH regeneration with glutamate dehydrogenase (GDH) and carbonic anhydrase	5.04	19.8	10	^58^
formate	FDH + GlyDH	modified natural zeolite	multienzymatic system with NADH regeneration by GlyDH (DHA not addressed)	5.4	123.8	--	^59^
DHA	GlyDH	silica nanoparticles (SNPs) poly(ethylene glycol) (PEG) hydrogels	dual immobilization strategy	1.7	12.6	7	^60^
DHA	GlyDH	agarose beads activated with glyoxyl groups and further cross-linked with dextran aldehyde	NADH regeneration by NADH oxidase (NOX)	3.0	33.8	1	
DHA	GlyDH	magnetic mesoporous silica	cross-linking with glutaraldehyde to prepare nanoscale enzyme reactors	0.18	0.02	7	^61^
glycerol carbonate	electrospin Li/Al nanofibers	–	reaction in ionic liquid, high pressure and temperature	11.45	98	6	^62^
glycerol carbonate	lipase from *Candida rugosa.*	octyl-agarose activated with divinyl sulfone	monophasic system with glycerol and ethylene carbonate at elevated temperatures	4500	2242	4	^63^

In this work, five batches were conducted, but the number could likely have been extended without substantially impacting productivity, potentially leading to an even higher biocatalyst yield. This highlights immobilization as a key tool in biocatalysis for reaction intensification, especially under harsh industrial conditions. Table S8 summarizes the performance metrics of the three synthesized molecules for the two addressed reaction conditions. After five reaction cycles, 1.77 and 1.49 g of formate were produced for the reaction with pure and crude substates, respectively, demonstrating an efficient alternative for CO_2_ bioreduction into high-value molecules such as formate.

When the environmental impact of this process was evaluated, an E-factor of 108.8 was obtained, considering the three compounds synthesized simultaneously in the one-pot system and the water of the aqueous media as waste. According to Domínguez de María,^54^ the obtained E-factor for this system is lower than the one expected for a conventional biocatalytic process conducted in aqueous media at larger industrial scales, where the reaction medium is not recycled (E-factor: 122). Compared with electrochemical, photochemical, and chemical CO_2_ reduction platforms, the energy data provided by several Life Cycle Assessments (LCAs) show a high generation of waste associated with the use of nonrecyclable organic solvents, critical metals (Ag, Pd, Ru, etc.), and the rapid degradation of their catalysts, as well as the use of electron donors mediators that are consumed in the reaction and generate waste.^55,56^ Further studies on this technology will provide the necessary data to perform an LCA, enabling a more comparable process analysis. However, the findings reported in this work highlight the environmental potential of enzymatic CO_2_ reduction systems as a sustainable alternative to conventional catalytic approaches.

For DHA, cumulative yields of 16 and 14 mg L^–1^ h^–1^ were obtained, respectively (Figure 5A). Compared to the reaction with free enzymes, the DHA productivity is similar in both reactions^19^ (not considering the adsorbed fraction). The catalyst yields were 64.1 and 59.4 mg g^–1^, respectively (Figure 5B). According to the literature, glutaraldehyde cross-linking of GlyDH is widely documented for glycerol oxidation in multicycle reactions, yielding DHA productivities similar or lower than this study.^42,60^ Thus, this platform presents a highly promising approach for the sustainable production of this high-value chemical through waste valorization.

Regarding GC, cumulative STY of 56.4 and 66.4 mg L^–1^ h^–1^ were obtained, respectively (Figure 5A). This slight difference is also observed in the catalyst performance under industrial conditions, attributed to a slight improvement in GC production under these conditions as previously discussed, achieving catalyst yields of 225.6 and 272.8 mg g^–1^, respectively (Figure 5B). This underscores the robustness of this biocatalyst for the synthesis of this high-value molecule. GC yields are typically much higher using metal catalysts, ionic liquids, and high temperatures and pressures.^62,64^ Enzymatic synthesis of this compound, primarily by lipases, also shows high productivity, but requires surfactants, high temperatures, and organic substrates instead of CO_2_.^63,65^ Despite this, the valorization of crude glycerol was successfully achieved, yielding two high-value-added molecules, DHA and glycerol carbonate. Likewise, the feasibility of this multienzymatic system for mitigating CO_2_ emissions was demonstrated through the remarkable production of formate and glycerol carbonate.

Regarding the separation of these high-value compounds, the sustainable isolation of formate through liquid–liquid extraction using a biorenewable solvent, 2-methyltetrahydrofuran (2-MTHF), has been previously reported as an effective strategy in the reaction with free enzymes.^19^ In the case of DHA, its selective separation via adsorption onto ion-exchange resins has also been explored.^66^ These methods highlight the potential for further downstream processing, supported by the easy separation of the coimmobilized biocatalyst.

## 4 Conclusions

The successful valorization of two abundant industrial waste products into three high-value-added compounds was performed in a one-pot multienzyme system from a CCU perspective. First, a bifunctional biocatalyst with both FDH and GlyDH enzymes was effectively prepared and optimized based on stability and activity through a one-step purification/coimmobilization strategy. Its application led to a marked intensification of the reaction, reaching an impressive formate concentration of 50.4 ± 0.3 mM (2.3 g L^–1^), which stands as the highest value reported to date for enzymatic catalysis. Likewise, glycerol valorization yielded DHA, with glycerol carbonate formed as a byproduct. To address the gap between bench-scale trials and industrial environment conditions, the performance of the biocatalyst was evaluated with crude substrates. The results demonstrated the feasibility of the system to address key bottlenecks in CCU research by using a bifunctional biocatalyst capable of intensifying the reaction, reducing inhibition, and improving stability and reusability over multiple reaction cycles. This sustainable system, aligned with the principles of circular economy, represents a remarkable strategy for the coproduction of valuable molecules, potential reduction of operational costs, and paves the way for exploring new methodologies for integrated product purification.

## Supplementary Material



## Data Availability

All data sets generated or analyzed during this study are available at 10.34810/data2184.

## Notes

Production and purification of the enzymes; quantification of substrates and products; carrier functionalization; calculations for biocatalyst preparation; study of different protein loading levels for immobilization; confocal microscopy analysis; adsorption isotherms; determination of half-life (*t*_1/2_) and IC_50_; experimental setup; composition of crude glycerol; FDH immobilization on different carriers; immobilization with pure enzymes and crude cell extracts; workflow of biocatalyst washing steps and corresponding SDS-PAGE; immobilization at different protein loadings; final bifunctional biocatalyst immobilization; confocal microscopy images; adsorption isotherms of each compound; study of GlyDH inhibition by DHA; mass balance of DHA adsorption; performance metrics of reaction cycles; and E-factor calculation (PDF)
