# Supplementary material for: Enzyme-Powered CO2 Utilization: A Bifunctional Immobilized Biocatalyst for Intensified CCU of Industrial Feedstocks to High-Value Chemicals
Source: ACS Sustain Chem Eng. 2025 Dec 24;14(1):86–98. doi: 10.1021/acssuschemeng.5c07343 (PMC12801976; doi:10.1021/acssuschemeng.5c07343)
Supplement: Supplementary file 1 [file sc5c07343_si_001.pdf]

<u>Supporting Information (SI)</u>

Enzyme-powered CO_2 utilization: A bifunctional immobilized biocatalyst for intensified CCU of industrial feedstocks to high-value chemicals.

Sady Roberto Rodriguez, Oscar Romero & Marina Guillén.*

Bioprocess Engineering and Applied Biocatalysis Group, Department of Chemical, Biological and Environmental Engineering, Universitat Autònoma de Barcelona, 08193 Bellaterra, Spain.

Number of pages: **26**

Figures: **10**

Tables: **8**

Contents

1. EXPERIMENTAL SECTION S2

1.1 Enzymes production and purification. S2

1.2 SDS-PAGE electrophoresis...S4

1.3 HPLC analysis...S5

1.4 NADH quantification S5

1.5 Carriers functionalization S6

1.6 Protein content by Bradford assay S6

1.7 Calculations for preparing the bifunctional co-immobilized biocatalyst from cell lysates. S6

1.8 Study of different protein loadings on Ni^{2+}-ReliZyme. S8

1.9 Confocal microscopy analysis of fluorophore-label proteins S8

1.10 Adsorption Isotherms S9

1.11 Half-life determination S9

1.12 Half maximal inhibitory concentration (IC_{50}) determination. S10

2. EXPERIMENTAL INFORMATION S11
3. RESULTS...S12

1. EXPERIMENTAL SECTION

1.1 Enzymes production and purification.

FDH from *Candida boidinii* and GlyDH from *Geobacillus stearothermophilus* were expressed in recombinant *Escherichia coli* BL21 (DE3) strains transformed in pet26b(+), with kanamycin resistance and 6xHis-tag at C-terminal. Briefly, a pre-inoculum was prepared by adding 100 μL of glycerol stocks to 25 mL of defined media (composition described by Vidal *et al.* [1]) and incubated overnight at 37 °C with 150 rpm agitation. Inoculum cultures were prepared twice with 200 mL of defined media, inoculated with pre-inoculum at an initial OD_{600} of 0.2 and incubated at 37 ^{0}C with 150 rpm agitation. When the inoculum reached an OD_{600} of 1.0, they were transferred to 2.5 L of defined media prepared in a 5 L bioreactor Applikon®. An initial batch-phase was performed at 30 °C, pH 7.0 and pO_2 30% adjusting the agitation between 450 and 1150 rpm and supplying air and/or pure oxygen at a flow of 1.5 L min^{-1} according to the biomass requirements. A 1:10 antifoam solution was used throughout the process. The pH was controlled with a NH_4OH 15% v/v and H_2SO_4 2 M solutions. When the glucose was completely consumed, the fed-batch phase was initiated with a preprogrammed exponential addition of feeding medium (composition defined by Vidal *et al.* [1]). The induction phase was started when an OD_{600} between 100 - 120 was reached, by the

addition of β-D-1-thiogalactopyranoside (IPTG) at a 0.1 mM final concentration. The induction phase was carried out for 4 hours.

The biomass obtained was centrifuged at 10,000 rpm for 15 minutes at 4 °C. *E. coli* cells expressing FDH enzyme were resuspended in phosphate buffer 100 mM and NaCl 100 mM (pH 7.0) and *E. coli* cells expressing GlyDH enzyme were resuspended in phosphate buffer 10 mM (pH 7.0). Cells suspensions were disrupted using a cell disruptor (Constant System Ltd.) followed by a centrifugation of the resulting cell lysate (10,000 rpm for 45 minutes at 4 °C).

Cell lysates were purified using an AKTA Pure 150 FPLC system (GE Healthcare®). Affinity chromatography was used for the purification of FDH enzyme, which consists of a column packed with agarose functionalized with nickel ions (Ni^{2+}). The column was initially equilibrated with phosphate buffer 100 mM and NaCl 100 mM (pH 7.0). This same buffer was used to load the sample and then wash the column before elution. Unbounded proteins were eluted during this step. FDH was eluted from the column by applying phosphate 100 mM, NaCl 100 mM and imidazole 300 mM buffer (pH 7.0). In the case of GlyDH, an ion exchange method was used with a column packed with diaminoethyl-agarose. Equilibration, sample loading and washing were carried out with 10 mM phosphate buffer (pH 7.0). Unbound material was washed out of the column using this buffer. GlyDH enzyme was eluted with a linear gradient using phosphate 100 mM + NaCl 300 mM buffer (pH 7.0). All the proteins were detected under 280 nm UV light. Catalytic activity and protein concentration were measured for each fraction obtained. Protein concentration was measured by Bradford's method [2] , using Bovine serum

albumin (BSA) as a standard. The samples containing FDH and GlyDH were dialyzed on a cellulose membrane using phosphate buffer 100 mM (pH 7.5) for 1 h, overnight, and then for 1 h more.

1.2 SDS-PAGE electrophoresis.

The molecular weights were estimated from the electrophoretic mobility of the proteins in a Sodium Dodecyl Sulfate Polyacrylamide Gel (SDS-PAGE). Samples were prepared at a concentration of 0.5 mg mL^{-1} and incubated in the loading buffer (Laemmli buffer 4x + β- mercaptoethanol 10:1) for 10 minutes at 95 °C. Subsequently, 15 µL of sample and 7 µL of standard proteins were loaded into the gel. Electrophoresis was performed for 80 minutes at 120 V. The gel was stained with Coomassie InstantBlue® and washed with water until blue bands appeared. The gel was analyzed using the Image Lab software from BIO-RAD.

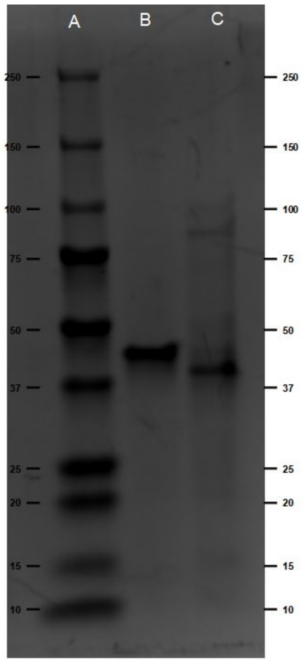

Figure S1. SDS-PAGE of purified Formate dehydrogenase (FDH) and Glycerol dehydrogenase (GlyDH) enzymes. (A) Standard proteins. (B) FDH enzyme (41 kDa [3]). (C) GlyDH enzyme (39.5 kDa [4])

1.3 HPLC analysis

Formate, DHA, and glycerol were quantified using an Agilent 1220 Infinity II liquid chromatograph with ion exchange chromatography, employing an IC-Sep COREGEL 87H3 column and a mobile phase of 0.5 mM sulfuric acid (H_2SO_4) and acetonitrile (65:35). The analysis was performed with a flow rate of 0.6 mL min^{-1}, 20 μL injection, column temperature of 35 °C, and detection at 210 nm using a UV/visible detector and at 35 °C with an RID detector. For glycerol 1,2-carbonate, reverse-phase chromatography was used with a C-18 CORTECS column and 0.004 N sulfuric acid as the mobile phase, a flow rate of 0.8 mL·min^{-1}, a 100 μL injection volume, and detection using an RID detector at 35 °C. Triplicate assays were performed.

1.4 NADH quantification

NADH was quantified by UV/visible spectrophotometry at 340 nm using the SPECTROstar microplate reader. Phosphate buffer 100 mM pH 7.5 was used as blank. Triplicate assays were performed.

1.5 Carriers functionalization

ReliZyme and Purolite resins were treated with a 2 M iminodiacetic acid solution (pH 11) at a 1:10 support-to-solution ratio and incubated overnight at 25°C [5] . After filtering and washing with water, the resins, as well as High Density Chelating agarose 6BCL, were functionalized with nickel ions using a 200 mM nickel sulfate solution at a 1:5 support-to-solution ratio, incubating for 2 hours at 25°C. The resins were then filtered, washed with water, and stored at 4°C.

1.6 Protein content by Bradford assay

Total protein concentration was determined using the Bradford assay on a SPECTROstar Nano plate reader at 595 nm. A calibration curve was generated with bovine serum albumin (BSA) standard in the range of 0–1.5 mg mL^{-1} . For the assay, 200 µL of Bradford reagent was mixed with 7 µL of the sample and incubated for 15 minutes. Triplicate assays were performed.

1.7 Calculations for preparing the bifunctional co-immobilized biocatalyst from cell lysates.

The preparation of the bifunctional co-immobilized biocatalyst was carried out based on the FDH:GlyDH ratio of 1:7, previously optimized using free enzymes [6] . Applying these calculations, the amount of each enzyme lysate to be applied to a given quantity of carrier can be determined. Notably, exclusive information from the cell (Eq. S4)

lysate must be considered, such as the recovered activity at low protein loading (2 mg total protein g^{-1} carrier), the expression level of each enzyme in the lysate as determined by SDS-PAGE electrophoresis, as well as the total protein concentration (mg mL^{-1}) and enzymatic activity (U mL^{-1}) in the lysate. A maximum loading capacity of 50 mg (combining FDH and GlyDH) per g of carrier was established. The following equations were applied sequentially to determine the volume (mL) of each enzyme lysate required for immobilization or co-immobilization:

$$UP = \frac{\text{Total protein concentration (mg mL}^{-1}\text{)}}{\text{Enzymatic activity (U mL}^{-1}\text{)}}$$

$$ME = \frac{\text{CAO x \% enzyme expression SDS-PAGE}}{\text{Recovered activity* (\%) x UP}}$$

$$XEL = \frac{50}{ME_{FDH} + ME_{GlyDH}}$$

$$MGL = ME \text{ x } XEL$$

$$MLL = \frac{MGL}{\text{Total protein concentration (mg mL}^{-1}\text{) x \% expression SDS-PAGE}}$$

(Eq. S5)

Where:

UP = Specific activity per mg total protein in the lysate (U mg^{-1})

CAO = Catalytic activity per mL optimized for the reaction using free enzymes: for FDH, 0.5 U mL^{-1}, and for GlyDH, 3.5 U mL^{-1} (as reported by Rodriguez *et al.* [6])

ME = Milligrams of enzyme per unit of activity required in the reaction (mg).

XEL = Number of times 1 gram of carrier can be loaded, considering the combined amount of both enzymes and a total capacity of 50 mg per gram of carrier.

MGL = Milligrams of enzyme (either FDH or GlyDH) per gram of carrier (mg).

MLL = Milliliters of lysate required to provide the necessary milligrams of enzyme per gram of carrier (mL).

* Recovered activity at low protein loading (2 mg total protein g^{-1} carrier), with the less diffusional restrictions observed.

1.8 Study of different protein loadings on Ni^{2+}-ReliZyme.

The maximum protein loading capacity of the Ni^{2+}-ReliZyme carrier was determined by individually applying different total protein amounts of each enzyme (GlyDH and FDH) from cell lysates. Immobilization experiments were conducted following protocols 2.3 and 2.5 in the manuscript. For each test, the enzyme solution was prepared to contain the specific amount of GlyDH or FDH required for the intended loading.

1.9 Confocal microscopy analysis of fluorophore-label proteins

The distribution of immobilized and co-immobilized fluorophore-labeled FDH and GlyDH was analyzed using a Zeiss LSM 980 confocal microscope (Carl Zeiss Microscopy GmbH, Oberkochen, Germany). FDH and GlyDH purified were labeled with Alexa Fluor™ 488 and 610-X dyes, respectively. Each enzyme was prepared in 100 mM

phosphate buffer (pH 7.5) in a 5 mg mL^{-1} concentration and labeled at a 1:5 enzyme-to-dye molar ratio for 1 h at room temperature with constant stirring. Labeled proteins were purified by centrifugation using an exclusion column packed with agarose (4000 rpm for 3 min) and then eluted by centrifugation (4000 rpm for 5 min). Samples were placed in glass-bottom dishes (Ibidi, GmbH, Gräfelfing, Germany) for visualization under the microscope. Excitation/emission conditions were 490/525 nm for Alexa Fluor™ 488 and 603/623 nm for Alexa Fluor™ 610-X. The images were acquired using a Plan-Apochromat 63×/1.40 Oil DIC M27 objective, and the obtained micrographs were analyzed in the ZEN software (Carl Zeiss Microscopy GmbH).

1.10 Adsorption Isotherms

Adsorption isotherms of formate, DHA, glycerol carbonate, and glycerol were evaluated on ReliZyme carrier and the bifunctional biocatalyst under non-reactive conditions. Formate, DHA, and glycerol carbonate concentrations ranged from 0 to 250 mM, while glycerol concentrations ranged from 0 to 500 mM. For the assay, 1 g of absorbent was incubated in 10 mL of each compound -prepared in 100 mM phosphate buffer (pH 7.0)- at 30 °C and under continuous stirring at 300 rpm until a stable concentration was reached.

1.11 Half-life determination

The half-life (t ½) of each enzyme was determined using the following equation, adapted from Yamane *et al.* [7]:

(Eq. S6)

$$t_{1/2} = t_1 + (50 - A_2)\frac{t_2 - t_1}{A_1 - A_2}$$

Where:

t_1 = Time when the relative activity remains above 50%.

t_2 = Time when the relative activity remains below 50%.

A_1 = Relative activity at time t_1

A_2 = Relative activity at time t_2

1.12 Half maximal inhibitory concentration (IC_{50}) determination.

The IC_{50} was calculated using the following equation:

$$IC_{50} = C_{X1} + \frac{(A_{X1} - 50)}{(A_{X1} - A_{X2})} \text{ x } (C_{X2} - C_{X1}) \qquad \text{(Eq. S7)}$$

Where:

IC_{50} = Half maximal inhibitory concentration

A_{X1} = Activity of the experimental point above 50% of relative activity

A_{X2} = Activity of the experimental point below 50% of relative activity

C_{X1} = Concentration of the experimental point above 50% of relative activity

C_{X2} = Concentration of the experimental point below 50% of relative activity

2. EXPERIMENTAL INFORMATION

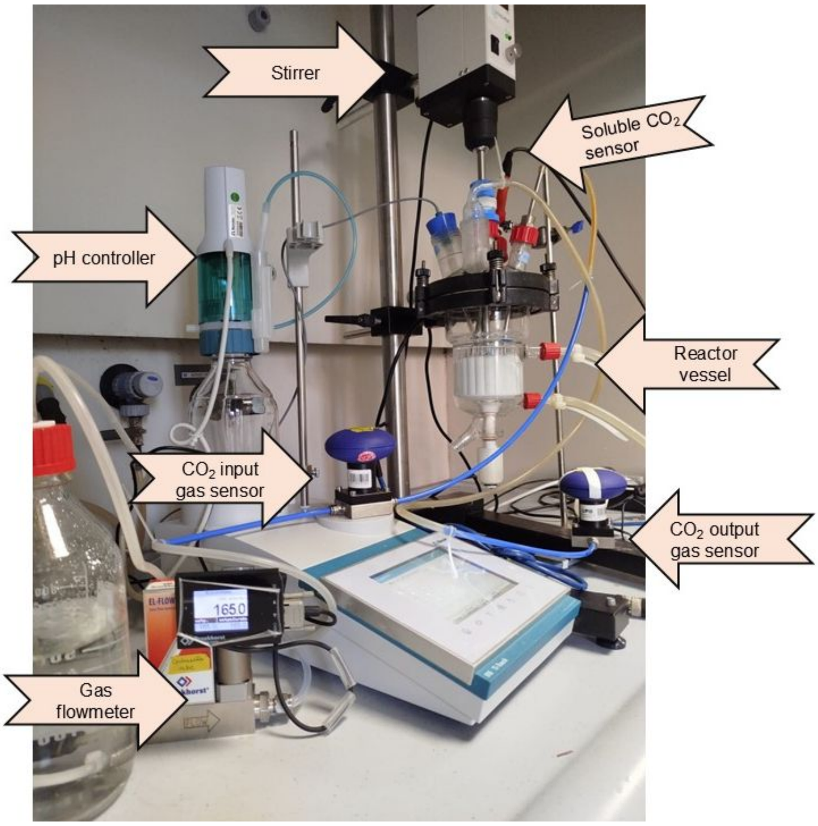

Figure S2. Experimental set-up for the multi-enzymatic CO_2 reduction to high-value compounds with continuous CO_2 supplementation.

Table S1. Data sheet of crude glycerol provided by ecoMotion Diesel S.A.

Analysis	Results (%)
Glycerol	64
Water	12
Matter Organic Non-Glycerol	13
Ashes	11.5
Methanol	0.04
pH	5.5

3. RESULTS

Table S2. Summary of the evaluation of different IMAC supports for FDH immobilization by offering 10 mg of purified protein per gram of carrier (25 U g^{-1}).

Parameters	Ni^{2+}-Agarose	EziG Coral	Ni^{2+}-ReliZyme	Ni^{2+}-Purolite
Offered activity (U g^{-1})	25.3 ± 0.2	25.8 ± 0.4	25.8 ± 0.1	24.9 ± 0.3
Observed biocatalyst activity (U g^{-1})	23.8 ± 0.3	18.4 ± 0.1	24.2 ± 0.3	21.8 ± 0.2
Yield - Activity (%)	99.8 ± 0.1	97.8 ± 0.2	100 ± 0.2	100 ± 0.1
Retained activity (%)	94.5 ± 0.3	73.7 ± 0.3	95.2 ± 0.2	89.2 ± 0.2
Recovery activity (%)	94.2 ± 0.1	71.5 ± 0.4	94.5 ± 0.1	87.7 ± 0.3
Bounded proteins (mg g^{-1})	10.4 ± 0.1	8.7 ± 0.2	10.1 ± 0.3	9.9 ± 0.2
Immobilization yield –Proteins (%)	100 ± 0.2	83.9 ± 0.2	100 ± 0.2	100 ± 0.1

Table S3. Summary of the parameters evaluated in the sequential co-immobilization of purified and cell lysates GlyDH and FDH. A protein load of 2 mg g^{-1} carrier of each enzyme was employed. *GC- GlyDH: GlyDH coated with glutaraldehyde*

Parameters	Purified		Cell lysate	
	GC-GlyDH	FDH	GC-GlyDH	FDH
Offered activity (U g^{-1})	3.1 ± 0.1	3.8 ± 0.2	3.3 ± 0.2	3.7 ± 0.1
Observed biocatalyst activity (U g^{-1})	1.5 ± 0.1	3.5 ± 0.1	1.4 ± 0.1	3.5 ± 0.2
Yield - Activity (%)	100 ± 0.2	100 ± 1.3	100 ± 2.1	100 ± 0.5
Retained activity (%)	51.1 ± 0.4	95.4 ± 1.3	48.7 ± 1.1	95.1 ± 0.6
Recovery activity (%)	50.3 ± 0.2* / 46.5 ± 0.3	95.1 ± 0.3	48.4 ± 0.7* / 43.9 ± 0.6	94.8 ± 0.8
Bounded proteins (mg g^{-1} carrier)	1.73 ± 0.2	1.98 ± 0.2	1.9 ± 0.2	2.2 ± 0.1
Yield – Proteins (%)	85.4 ± 0.5	100 ± 1.4	21.3 ± 0.8	48.5 ± 0.6

**GC-GlyDH recovery activity before FDH addition.*

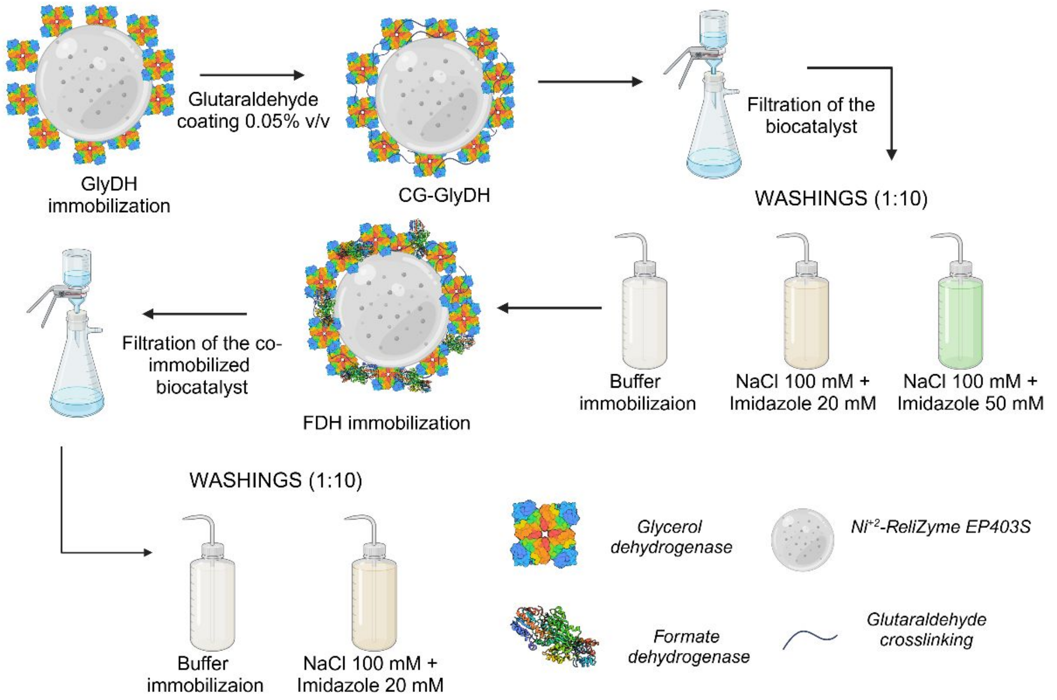

Figure S3. Workflow of the co-immobilization process of GC-GlyDH and FDH from cell lysates onto the Ni^{2+}-ReliZyme carrier. Washings were performed at a 1:10 ratio (10 mL of each solution per gram of carrier).

Brief discussion: Imidazole, a structural analog of the histidine imidazole ring, can effectively reduce nonspecific binding at low concentrations (5–50 mM) without significantly affecting His-tagged proteins [8] . Additionally, sodium chloride can reduce ionic interactions between nonspecific proteins and the carrier.

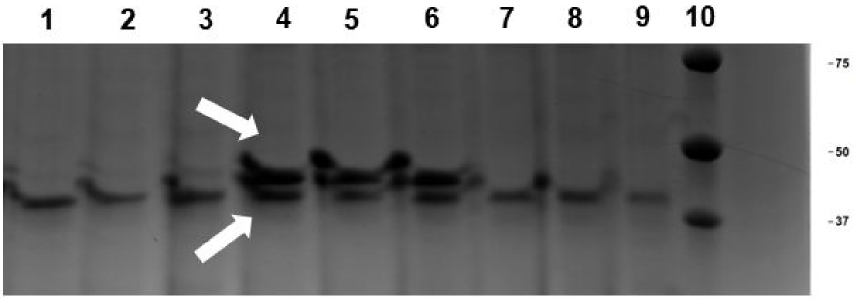

Figure S4. SDS-PAGE electrophoresis gel of GlyDH (39.5 kDa [4]) and FDH (41 kDa [3]) co-immobilized on the Ni^{2+}-ReliZyme carrier. The biocatalyst was washed with different solutions as follows: GC-GlyDH immobilization: (1) wash with immobilization buffer, (2) wash with 100 mM NaCl + 20 mM imidazole, (3) wash with 100 mM NaCl + 50 mM imidazole. (4, 5, 6) Final bifunctional biocatalyst (GC-GlyDH + FDH): (7) wash with immobilization buffer, (8) wash with 100 mM NaCl + 20 mM imidazole, (9) wash with 100 mM NaCl + 50 mM imidazole. (10) Proteins standard.

Brief discussion: GlyDH is slightly displaced from the biocatalyst in all washings, with a loss of approximately 9.4 ± 0.7 % of the initial enzyme load. In contrast, FDH showed a minimal loss of only 1.8 ± 0.2 % in the two washings applied. These values were considered when preparing subsequent biocatalysts.

Tables S4 and S5. Summary of evaluated parameters for the immobilization of different GlyDH and FDH loadings from cells lysate onto Ni^{2+}-ReliZyme.

Parameters	Theorical mg GlyDH g^{-1} carrier offered						
	0.5	2	5	10	25	50	75
Offered activity (U g^{-1})	1.7 ± 0.1	6.5 ± 0.1	17.4 ± 0.4	33.1 ± 0.1	87.5 ± 0.4	165.2 ± 0.4	248.4 ± 0.5
Theorical biocatalyst activity (U g^{-1})	1.0 ± 0.1	3.9 ± 0.1	10.6 ± 0.3	20.2 ± 0.2	53.3 ± 0.6	100.6 ± 0.2	151.3 ± 0.5
Observed biocatalyst activity (U g^{-1})	1.0 ± 0.1	3.7 ± 0.2	7.4 ± 0.5	11.7 ± 0.3	13.6 ± 0.3	14.0 ± 0.5	13.9 ± 0.2
Yield - Activity (%)	100 ± 0.4	100 ± 0.3	100 ± 0.1	100 ± 0.2	100 ± 0.1	98.5 ± 0.1	93.5 ± 0.4
Retained activity (%)	61.3 ± 0.3	59.5 ± 0.6	45.3 ± 0.5	37.8 ± 0.3	22.3 ± 0.5	8.7 ± 0.4	6.5 ± 0.2
Recovery activity (%)	60.9 ± 0.1	56.3 ± 0.3	42.3 ± 0.3	35.3 ± 0.7	15.5 ± 0.3	8.5 ± 0.3	5.6 ± 0.3
Bounded proteins (mg g^{-1})	0.6 ± 0.1	1.9 ± 0.1	4.8 ± 0.6	9.7 ± 0.1	24.3 ± 0.2	48.9 ± 0.2	65.1 ± 0.3
Yield – Proteins (%)	22.3 ± 0.5	22.5 ± 0.3	21.4 ± 0.2	22.2 ± 0.4	21.7 ± 0.1	19.1 ± 0.1	17.1 ± 0.1

Parameters	Theorical mg FDH g^{-1} carrier offered						
	0.5	2	5	10	25	50	75
Offered activity (U g^{-1})	0.6 ± 0.1	2.2 ± 0.2	12.3 ± 0.4	23.5 ± 0.2	54.1 ± 0.7	107.4 ± 0.1	160.3 ± 0.8
Theorical biocatalyst activity (U g^{-1})	0.6 ± 0.1	2.2 ± 0.2	12.3 ± 0.4	23.5 ± 0.2	54.0 ± 0.1	107.2 ± 0.1	159.8 ± 0.8
Observed biocatalyst activity (U g^{-1})	0.6 ± 0.1	2.2 ± 0.3	11.9 ± 0.5	22.2 ± 0.7	46.7 ± 0.6	79.7 ± 0.6	88.6 ± 0.9
Yield - Activity (%)	100.0 ± 0.3	100.0 ± 0.4	100.0 ± 0.1	100.0 ± 0.2	100.0 ± 0.3	100.0 ± 0.3	96.5 ± 0.5
Retained activity (%)	100.0 ± 0.3	100.0 ± 0.4	99.2 ± 0.3	95.7 ± 0.2	88.3 ± 0.2	74.7 ± 0.2	58.1 ± 0.3
Recovery activity (%)	99.8 ± 0.1	99.5 ± 0.1	97.1 ± 0.4	94.5 ± 0.4	86.4 ± 0.3	74.2 ± 0.2	55.3 ± 0.4

Bounded proteins (mg g^{-1})	0.5 ± 0.1	2.2 ± 0.4	5.1 ± 0.1	10.2 ± 0.1	24.8 ± 0.1	48.1 ± 0.3	68.1 ± 0.3
Yield – Proteins (%)	52.4 ± 0.4	51.6 ± 0.3	51.2 ± 0.5	51.3 ± 0.2	50.6 ± 0.2	49.8 ± 0.2	46.7 ± 0.1

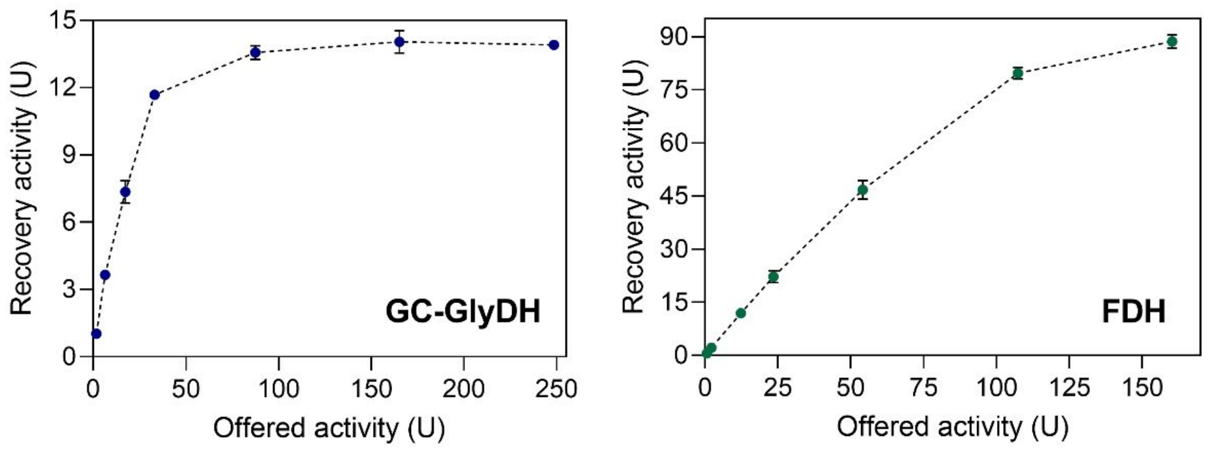

Figure S5. Study of different loadings of GC-GlyDH and FDH in immobilization on Ni^{2+}-ReliZyme, comparing the offered activity versus the recovered activity.

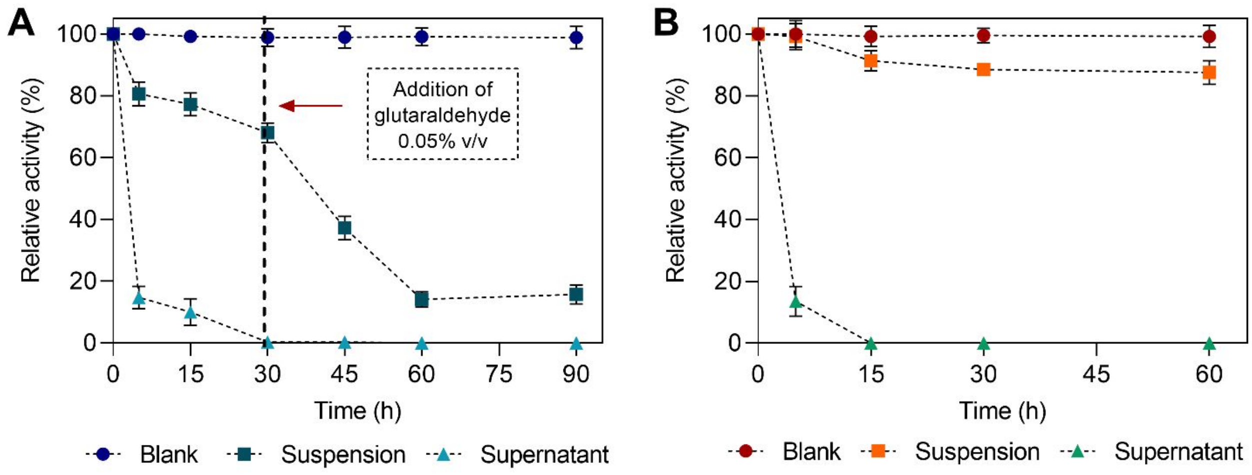

Figure S6. Sequential co-immobilization of cell lysates GlyDH and FDH onto Ni^{2+}-ReliZyme carrier. The relative activity was calculated by considering the activity offered for each enzyme as 100%. (A) GC-GlyDH. (B) FDH.

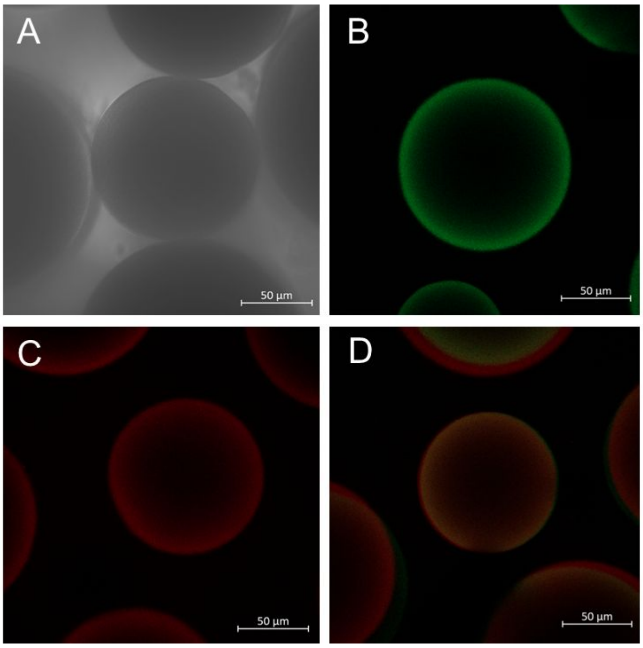

Figure S7. Compilation of confocal microscopy images of the bifunctional biocatalyst GC-GlyDH and FDH on Ni^{2+}-ReliZyme carrier. Images were acquired using a Zeiss LSM 980 microscope, with FDH and GlyDH labeled with Alexa Fluor™ 488 and Alexa Fluor™ 610-X, respectively. Excitation/emission parameters were 490/525 nm for Alexa Fluor™ 488 and 603/623 nm for Alexa Fluor™ 610-X. (A) Ni^{2+}-ReliZyme carrier. (B) FDH. (C) GC-GlyDH. (D) Bifunctional biocatalyst (GC-GlyDH + FDH).

Table S6. Parameters evaluated in the adsorption isotherms of formate, DHA, glycerol carbonate and glycerol on Ni^{2+}-ReliZyme resin and the bifunctional biocatalyst according to Langmuir model.

Compound	Ni^{2+}-ReliZyme		Bifunctional biocatalyst	
	Q_{max}* $(mg\ g^{-1})$	K_L** $(mL\ mg^{-1})$	Q_{max} $(mg\ g^{-1})$	K_L $(mL\ mg^{-1})$
Formate	1.5 ± 0.1	0.245 ± 0.04	15.2 ± 0.2	0.182 ± 0.01

DHA	62.6 ± 0.3	0.111 ± 0.03	140.8 ± 0.5	0.048 ± 0.001
Glycerol carbonate	42.8 ± 0.4	0.130 ± 0.03	57.2 ± 0.4	0.042 ± 0.001
Glycerol	2.9 ± 0.1	0.381 ± 0.05	2.2 ± 0.2	0.316 ± 0.01

*Q_{max}: Maximum adsorption capacity. **K_L: Langmuir adsorption constant.

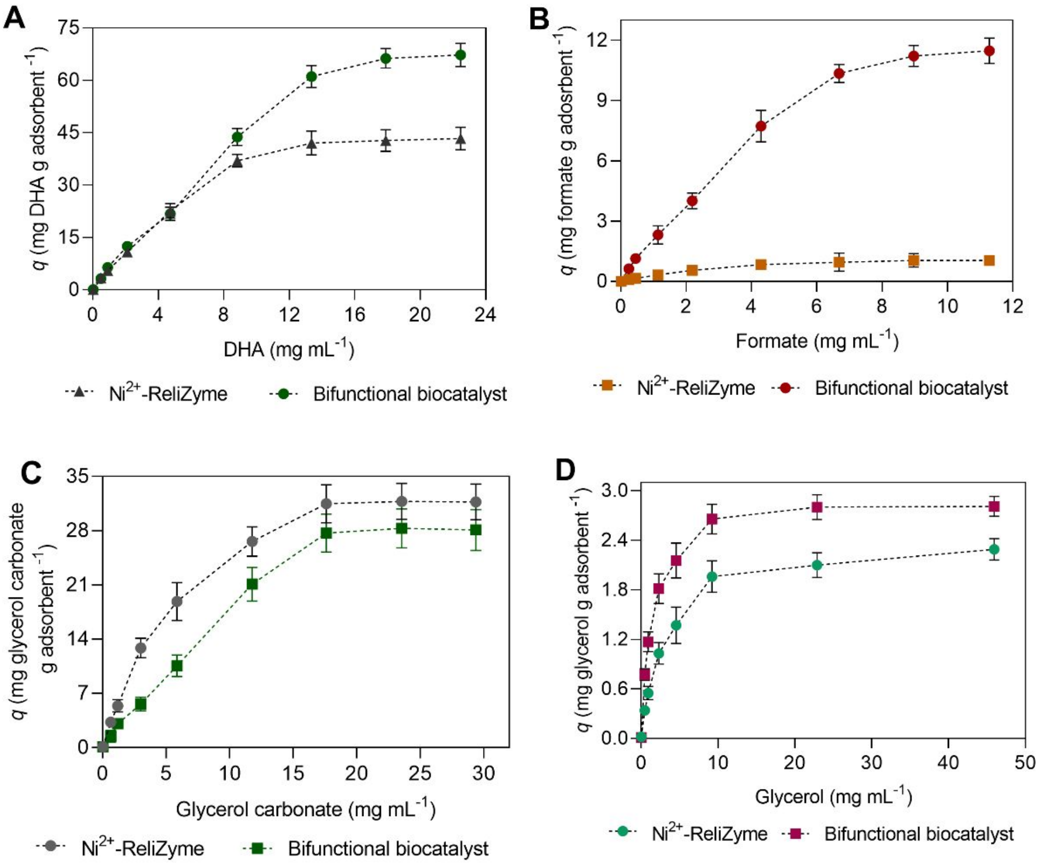

Figure S8. Adsorption isotherm of the compounds of multi-enzymatic system on Ni^{2+}-ReliZyme carrier and bifunctional biocatalyst (GC-GlyDH and FDH). (A) DHA. (B) Formate. (C) Glycerol carbonate. (D) Glycerol.

Brief discussion: Formate showed lower affinity for both adsorbents, remaining mainly in the aqueous phase due to its polarity. At pH 6.5 - 7.5, its deprotonated form may cause electrostatic repulsion with the carboxyl groups, limiting adsorption. However, several studies have shown effective formate adsorption on strong anionic resins due to interactions between the quaternary amine groups and the deprotonated carboxyl group

of formate [9], [10], [11]. The high reactivity of DHA may compete for adsorption, reducing the resin's capacity to bind formate. Glycerol carbonate showed a moderate affinity for both adsorbents (42.8 ± 0.4 and 57.2 ± 0.4 mg g^{-1}, respectively). Although it is a polar molecule due to the hydrophilic nature of its carbonyl group and glycerol backbone, the reduced electron density of its carbonate ring imparts an apolar character, allowing it to interact with hydrophobic surfaces such as the immobilization carrier. Finally, glycerol was the least reactive molecule for both adsorbents. At neutral pH, it has no net charge, resulting in minimal interaction with ionic surfaces. However, its adsorption on polar or non-ionic surfaces typically requires high concentrations, elevated temperatures, or basic pH for efficient adsorption [12], [13] .

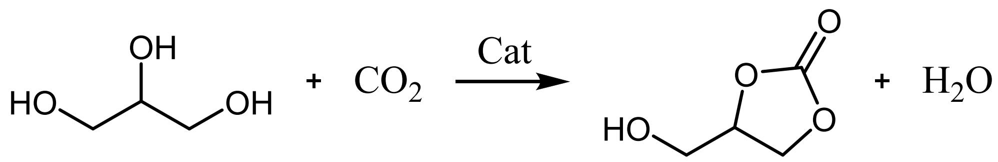

Figure S9. Direct carboxylation reaction between glycerol and CO_2 to produce glycerol 1,2-carbonate and water.

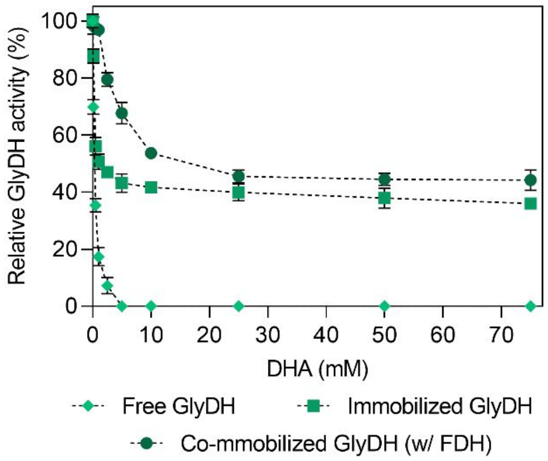

Figure S10. Inhibition study of the GlyDH enzyme by DHA in its free, immobilized, and co-immobilized (with FDH) forms on the Ni^{2+}-ReliZyme carrier. The 100% of relative activity was defined as the activity at 0 mM DHA concentration.

Table S7. Mass balance of the DHA produced and adsorbed onto the bifunctional biocatalyst over five consecutive reaction cycles.

**Reaction cycle**	**Formate concentration (mM)**	**DHA concentration (mM)***	**Total DHA produced (mg)****	**DHA in the medium (mg)**	**DHA adsorbed (mg)**	**DHA adsorbed (mg g $^{-1}$ biocatalyst)**
Reaction with pure substrates						
1	50.4 ± 0.3	12.2 ± 0.4	907.8 ± 1.9	218.0 ± 3.5	689.8 ± 2.2	34.5 ± 1.5
2	42.7 ± 1.2	13.2 ± 1.3	748.5 ± 2.4	232.1 ± 3.2	516.4 ± 2.1	26.5 ± 1.3
3	39.2 ± 2.1	15.5 ± 1.2	676.9 ± 3.2	267.0 ± 1.7	409.9 ± 1.9	21.4 ± 1.2
4	36.8 ± 0.3	16.6 ± 0.1	629.4 ± 2.1	284.1 ± 1.4	345.4 ± 1.4	18.2 ± 1.9
5	35.3 ± 1.2	17.3 ± 0.6	571.3 ± 2.5	279.6 ± 3.2	291.8 ± 1.9	15.8 ± 1.3
TOTAL					**2253.3 ± 3.5**	**116.3 ± 1.8**
Reaction with crude substrates						
1	43.3 ± 1.3	9.2 ± 0.2	779.2 ± 2.6	166.7 ± 1.7	612.4 ± 1.4	30.6 ± 1.2
2	35.7 ± 2.4	12.2 ± 1.1	633.1 ± 3.4	217 ± 1.5	416.1 ± 2.1	21.1 ± 1.4
3	32.3 ± 1.4	13.5 ± 0.7	566.8 ± 1.9	237 ± 3.2	329.8 ± 1.1	16.9 ± 1.8
4	30.4 ± 1.2	15.6 ± 0.9	526.0 ± 4.3	268.8 ± 1.1	257.2 ± 1.9	13.4 ± 2.0
5	28.3 ± 2.1	17.4 ± 0.5	484.6 ± 3.2	298.2 ± 4.5	186.5 ± 2.1	9.8 ± 1.9
TOTAL					**1802.0 ± 2.2**	**91.9 ± 2.1**

**Concentration of DHA quantified in the reaction medium. **Amount of DHA produced based on the synthesized formate concentration.*

Table S8. Performance metrics over five consecutive cycles for the multi-enzymatic synthesis of formate, DHA and glycerol carbonate (as a byproduct) from the reaction with pure substrates (gas mixture 24% CO_2 and pure glycerol) and the crude substrates (synthetic gas mixture from iron and steel industry + crude glycerol). **DHA yields were calculated based on formate production in the cycle 1.*

Reaction cycle	Concentration (mM)	STY* (mg L^{-1} h^{-1})	Specific productivity (mg L^{-1} h^{-1} g^{-1} or mg^{-1})		Catalyst yield (mg g^{-1} or mg^{-1})	
			Per catalyst amount (g)	Per total immobilized protein (mg) **	Per catalyst amount(g)	Per total immobilized protein (mg) **
Reaction with pure substrates						
Formate						
1	50.4 ± 0.3	28.4 ± 0.4	1.42 ± 0.09	0.028 ± 0.002	22.7 ± 0.8	0.45 ± 0.08
2	42.7 ± 1.2	24.0 ± 0.6	1.23 ± 0.1	0.024 ± 0.003	19.2 ± 1.9	0.38 ± 0.09
3	39.2 ± 2.1	22.0 ± 1.3	1.15 ± 0.06	0.023 ± 0.004	17.6 ± 1.5	0.35 ± 0.08
4	36.8 ± 0.3	20.7 ± 1.1	1.09 ± 0.2	0.022 ± 0.002	16.6 ± 1.3	0.33 ± 0.05
5	35.3 ± 1.2	19.8 ± 0.4	1.07 ± 0.1	0.021 ± 0.006	15.4 ± 0.3	0.31 ± 0.01
Cumulative		**22.1 ± 0.7**			**88.4 ± 1.8**	**1.75 ± 0.1**
DHA						
1	12.2 ± 0.4	13.7 ± 0.1	0.68 ± 0.04	0.014 ± 0.002	10.9 ± 0.9	0.22 ± 0.09
2	13.2 ± 1.3	14.9 ± 0.5	0.76 ± 0.1	0.015 ± 0.003	11.9 ± 0.5	0.24 ± 0.05

Reaction cycle	Concentration (mM)	STY* (mg L^{-1} h^{-1})	Specific productivity (mg L^{-1} h^{-1} g^{-1} or mg^{-1})		Catalyst yield (mg g^{-1} or mg $^{-1}$)	
			Per catalyst amount (g)	Per total immobilized protein (mg) **	Per catalyst amount(g)	Per total immobilized protein (mg) **
3	15.5 ± 1.2	17.4 ± 0.8	0.91 ± 0.09	0.018 ± 0.002	13.9 ± 1.6	0.28 ± 0.03
4	16.6 ± 0.1	18.7 ± 0.5	0.98 ± 0.05	0.019 ± 0.001	15.0 ± 1.9	0.30 ± 0.01
5	17.3 ± 0.6	19.4 ± 0.9	1.05 ± 0.1	0.021 ± 0.003	15.1 ± 2.3	0.30 ± 0.06
Cumulative		**16.0 ± 0.8**			**64.1 ± 1.6**	**1.27 ± 0.1**
Glycerol carbonate						
1	40.7 ± 0.2	60.1 ± 2.3	3.00 ± 0.1	--	48.0 ± 2.9	--
2	39.5 ± 2.1	58.3 ± 1.3	2.99 ± 0.6	--	46.7 ± 3.3	--
3	40.2 ± 1.3	59.3 ± 3.1	3.09 ± 0.8	--	47.4 ± 1.3	--
4	39.2 ± 1.3	57.9 ± 1.2	3.05 ± 0.4	--	46.3 ± 2.8	--
5	40.1 ± 0.5	59.1 ± 0.1	3.19 ± 0.2	--	46.0 ± 2.3	--
Cumulative		**56.4 ± 0.9**			**225.6 ± 2.4**	
Reaction with crude substrates						
Formate						
1	43.3 ± 1.3	22.9 ± 2.3	1.15 ± 0.1	0.023 ± 0.003	19.5 ± 0.9	0.39 ± 0.06
2	35.7 ± 2.4	18.9 ± 1.3	0.96 ± 0.2	0.019 ± 0.006	16.1 ± 2.3	0.32 ± 0.07

Reaction cycle	Concentration (mM)	STY* (mg L^{-1} h^{-1})	Specific productivity (mg L^{-1} h^{-1} g^{-1} or mg^{-1})		Catalyst yield (mg g^{-1} or mg^{-1})	
			Per catalyst amount (g)	Per total immobilized protein (mg) **	Per catalyst amount(g)	Per total immobilized protein (mg) **
3	32.3 ± 1.4	17.1 ± 0.9	0.88 ± 0.06	0.018 ± 0.001	14.5 ± 2.9	0.29 ± 0.1
4	30.4 ± 1.2	16.1 ± 0.7	0.84 ± 0.1	0.017 ± 0.001	13.7 ± 0.8	0.28 ± 0.08
5	28.3 ± 2.1	15.0 ± 0.6	0.79 ± 0.04	0.016 ± 0.002	12.8 ± 0.7	0.26 ± 0.03
Cumulative		**17.6 ± 0.3**			**74.8 ± 1.9**	**1.50 ± 0.3**
			DHA			
1	9.2 ± 0.2	9.8 ± 0.2	0.49 ± 0.05	0.010 ± 0.001	8.3 ± 2.6	0.17 ± 0.08
2	12.2 ± 1.1	13.0 ± 0.9	0.66 ± 0.02	0.013 ± 0.002	11.0 ± 1.2	0.22 ± 0.06
3	13.5 ± 0.7	14.3 ± 0.4	0.73 ± 0.03	0.015 ± 0.001	12.2 ± 1.7	0.24 ± 0.05
4	15.6 ± 0.9	16.5 ± 0.3	0.86 ± 0.09	0.017 ± 0.001	14.0 ± 2.9	0.28 ± 0.09
5	17.4 ± 0.5	18.5 ± 0.1	0.97 ± 0.1	0.020 ± 0.003	15.7 ± 2.5	0.32 ± 0.03
Cumulative		**14.0 ± 0.5**			**59.4 ± 2.5**	**1.19 ± 0.3**
			Glycerol carbonate			
1	50.6 ± 0.9	70.2 ± 0.9	3.51 ± 0.2	--	59.7 ± 0.5	--
2	48.3 ± 0.7	67.1 ± 1.6	3.41 ± 0.1	--	57.0 ± 2.3	--
3	47.8 ± 1.4	66.4 ± 2.3	3.41 ± 0.2	--	56.4 ± 1.1	--

Reaction cycle	Concentration (mM)	STY* (mg L^{-1} h^{-1})	Specific productivity (mg L^{-1} h^{-1} g^{-1} or mg^{-1})		Catalyst yield (mg g^{-1} or mg $^{-1}$)	
			Per catalyst amount (g)	Per total immobilized protein (mg) **	Per catalyst amount(g)	Per total immobilized protein (mg) **
4	45.5 ± 0.8	63.2 ± 2.5	3.29 ± 0.05	--	53.7 ± 2.3	--
5	44.9 ± 0.4	62.4 ± 1.0	3.28 ± 0.1	--	53.0 ± 1.9	--
Cumulative		**64.2 ± 1.3**			**272.8 ± 2.8**	

**STY: Space-time yield. **Total protein amount immobilized on the carrier (CG-GlyDH + FDH).*

Determination of Environmental factor (E-factor)

The E-factor was calculated according to the equation proposed by Sheldon [14]:

$$\text{E-factor} = \frac{\text{mass of waste generated (kg)}}{\text{mass of product obtained (kg)}} \qquad \text{(Eq. S7)}$$

In this work the calculation was carried out as follows:

Total reaction volume: 200 mL ≈ 0.2 kg (Considering the reaction medium [phosphate buffer], which is not reused)

Biocatalyst (reusable) = 0.02 kg

Product concentrations (first cycle):

- Formate= 2.3 g L^{-1} (equivalent to 0.00046 kg)
- DHA = 1.1 g L^{-1} (equivalent to 0.00022 kg) [not adsorbed fraction]
- Glycerol Carbonate = 4.8 g L^{-1} (equivalent to 0.00096 kg)

Total products = 0.00164 kg

$$\text{E-factor} = \frac{(0.2 - 0.02 - 0.00164)\ \text{kg}}{0.00164\ \text{kg}} = \mathbf{108.8}$$

REFERENCES

[1] L. Vidal, J. Pinsach, G. Striedner, G. Caminal, and P. Ferrer, 'Development of an antibiotic-free plasmid selection system based on glycine auxotrophy for recombinant protein overproduction in Escherichia coli', *J Biotechnol*, vol. 134, no. 1–2, pp. 127–136, 2008, doi: 10.1016/j.jbiotec.2008.01.011.

[2] M. M. Bradford, 'A rapid and sensitive method for the quantitation of microgram quantities of protein utilizing the principle of protein-dye binding', *Anal Biochem*, vol. 72, no. 1–2, pp. 248–254, 1976, doi: 10.1016/0003-2697(76)90527-3.

[3] N. E. Labrou, 'Improved purification of Candida boidinii formate dehydrogenase', *Bioseparation*, vol. 9, no. 2, pp. 99–104, 2000, doi: 10.1023/A:1008131320571.

[4] S. N. Ruzheinikov *et al.*, 'Glycerol Dehydrogenase: Structure, Specificity, and Mechanism of a Family III Polyol Dehydrogenase', *Structure*, vol. 9, no. 9, pp. 789–802, 2001, doi: 10.1016/S0969-2126(01)00645-1.

[5] C. Mateo, V. Grazu, J. M. Palomo, F. Lopez-Gallego, R. Fernandez-Lafuente, and J. M. Guisan, 'Immobilization of enzymes on heterofunctional epoxy supports', *Nat Protoc*, vol. 2, no. 5, pp. 1022–1033, May 2007, doi: 10.1038/nprot.2007.133.

[6] S. R. Rodriguez, G. Álvaro, M. Guillén, and O. Romero, 'Multienzymatic Platform for Coupling a CCU Strategy to Waste Valorization: CO_2 from the Iron and Steel Industry and Crude Glycerol from Biodiesel Production', *ACS Sustain Chem Eng*, vol. 13, no. 4, pp. 1440–1449, Jan. 2025, doi: 10.1021/acssuschemeng.4c04908.

[7] T. Yamane, P. Sirirote, and S. Shimizu, 'Evaluation of half-life of immobilized enzyme during continuous reaction in bioreactors: A theoretical study', *Biotechnol. Bioeng.*, vol. 30, no. 8, pp. 963–969, Dec. 1987, doi: 10.1002/bit.260300807.

[8] E. Sulkowski, 'Purification of proteins by IMAC', *Trends Biotechnol*, vol. 3, no. 1, pp. 1–7, Jan. 1985, doi: 10.1016/0167-7799(85)90068-X.

[9] M. A. Hoque, K. Abad, P. Kharel, A. Omosebi, and J. Thompson, 'Downstream Separation of Formic Acid with Anion-Exchange Resin from Electrocatalytic Carbon Dioxide ($COCO_2$) Conversion: Adsorption, Kinetics, and Equilibrium Modeling', *Ind Eng Chem Res*, vol. 63, no. 6, pp. 2779–2790, Feb. 2024, doi: 10.1021/acs.iecr.3c02946.

[10] B. W. Jeon, U. T. Phan, Y. Heo, H. H. Lee, J. Ryu, and Y. H. Kim, 'Enzymatic conversion of CO_2 to formate: The potential of tungsten-containing formate dehydrogenase in flow reactor system', *Journal of CO_2 Utilization*, vol. 82, Apr. 2024, doi: 10.1016/j.jcou.2024.102754.

[11] X. Lin *et al.*, 'Sorption behavior and mechanism investigation of formic acid removal by sorption using an anion-exchange resin', *Desalination Water Treat*, vol. 57, no. 1, pp. 366–381, Jan. 2016, doi: 10.1080/19443994.2014.966761.

[12] N. F. Jariah, M. A. Hassan, Y. H. Taufiq-Yap, and A. M. Roslan, 'Technological advancement for efficiency enhancement of biodiesel and residual glycerol refining: A mini review', *Processes*, vol. 9, no. 7, p. 1198, Jul. 2021, doi: 10.3390/pr9071198.

[13] T. Attarbachi, M. D. Kingsley, and V. Spallina, 'New trends on crude glycerol purification: A review', *Fuel*, vol. 340, May 2023, doi: 10.1016/j.fuel.2023.127485.

[14] R. A. Sheldon, 'Metrics of Green Chemistry and Sustainability: Past, Present, and Future', *ACS Sustain Chem Eng*, vol. 6, no. 1, pp. 32–48, Jan. 2018, doi: 10.1021/acssuschemeng.7b03505.
